# Synthesis and Biological Evaluation of Carvacrol-Based Derivatives as Dual Inhibitors of *H. pylori* Strains and AGS Cell Proliferation

**DOI:** 10.3390/ph13110405

**Published:** 2020-11-19

**Authors:** Francesca Sisto, Simone Carradori, Paolo Guglielmi, Carmen Beatrice Traversi, Mattia Spano, Anatoly P. Sobolev, Daniela Secci, Maria Carmela Di Marcantonio, Entela Haloci, Rossella Grande, Gabriella Mincione

**Affiliations:** 1 Department of Biomedical, Surgical and Dental Sciences, University of Milan, 20122 Milan, Italy; francesca.sisto@unimi.it; 2 Department of Pharmacy, “G. d’Annunzio” University of Chieti-Pescara, Via dei Vestini 31, 66100 Chieti, Italy; cbtraversi@gmail.com (C.B.T.); rossella.grande@unich.it (R.G.); 3 Department of Chemistry and Technology of Drugs, Sapienza University of Rome, P.le A. Moro 5, 00185 Rome, Italy; paolo.guglielmi@uniroma1.it (P.G.); mattia.spano@uniroma1.it (M.S.); daniela.secci@uniroma1.it (D.S.); 4 Institute for Biological Systems, “Annalaura Segre” Magnetic Resonance Laboratory, CNR, 00015 Monterotondo (Rome), Italy; anatoly.sobolev@cnr.it; 5 Department of Medical, Oral, and Biotechnological Sciences, “G. d’Annunzio” University of Chieti-Pescara, 66100 Chieti, Italy; dimarcantonio@unich.it (M.C.D.M.); gabriella.mincione@unich.it (G.M.); 6 Department of Pharmacy, University of Medicine, Tirana, Rr. Dibres 369, 1001 Tirana, Albania; entela.haloci@umed.edu.al

**Keywords:** carvacrol, *Helicobacter pylori*, AGS cells, semi-synthesis, drug resistance, dual agent, coumarin

## Abstract

This study reports on the synthesis, structural assessment, microbiological screening against several strains of *H. pylori* and antiproliferative activity against human gastric adenocarcinoma (AGS) cells of a large series of carvacrol-based compounds. Structural analyses consisted of elemental analysis, ^1^H/^13^C/^19^F NMR spectra and crystallographic studies. The structure-activity relationships evidenced that among ether derivatives the substitution with specific electron-withdrawing groups (CF_3_ and NO_2_) especially in the para position of the benzyl ring led to an improvement of the antimicrobial activity, whereas electron-donating groups on the benzyl ring and ethereal alkyl chains were not tolerated with respect to the parent compound (MIC/MBC = 64/64 µg/mL). Ester derivatives (coumarin-carvacrol hybrids) displayed a slight enhancement of the inhibitory activity up to MIC values of 8–16 µg/mL. The most interesting compounds exhibiting the lowest MIC/MBC activity against *H. pylori* (among others, compounds **16** and **39** endowed with MIC/MBC values ranging between 2/2 to 32/32 µg/mL against all the evaluated strains) were also assayed for their ability to reduce AGS cell growth with respect to 5-Fluorouracil. Some derivatives can be regarded as new lead compounds able to reduce *H. pylori* growth and to counteract the proliferation of AGS cells, both contributing to the occurrence of gastric cancer.

## 1. Introduction

Carvacrol is a naturally occurring monoterpene phenol abundant in several medicinal plants (especially within the Labiatae and Apiaceae families) which, besides its odoriferous and flavoring function, exhibits antimicrobial, food preserving, antioxidant and anticancer activities [1,2]. More in detail, carvacrol and its derivatives were shown to exert an interesting antimicrobial and antibiofilm effects against a large panel of Gram-positive and Gram-negative bacteria and fungi [3,4,5]. Its mechanism of action, albeit not yet fully elucidated, could involve structural and functional alterations of the membrane, dysregulation of nucleic acids, altered metabolism and ATP production.

Carvacrol and carvacrol-producing plants (*Satureja* spp., *Thymus* spp., and *Origanum* spp.) were also studied for their ability to inhibit *Helicobacter pylori* (*H. pylori*) growth, evidencing the possibility to introduce chemical modifications of this lead compound to improve this biological activity and/or to enhance its poor pharmacokinetic profile [6].

*H. pylori*, a microaerophilic Gram-negative bacterium, colonizes about the 50% of the world’s population representing the causative agent of the development of chronic gastritis, peptic ulcer and gastric cancer [7]; for the latter, it has been recognized as a class I carcinogen by the World Health Organization. Gastric cancer represents the third most common cancer worldwide [7,8] and epidemiological studies demonstrated that the eradication of *H. pylori* induces a decrease of incidence of such malignancy [9]. The triple therapy, consisting of a proton-pump inhibitor (PPI) and two different antimicrobial drugs, represented the anti-*H. pylori* standard therapy for the last 20 years. The failure of the above-mentioned therapy may be due to an increased antibiotic resistance to clarithromycin [10] and levofloxacin, two of the antimicrobials used in the triple therapy. In particular, levofloxacin was introduced a decade ago as an alternative to clarithromycin [11]. Recently, a bismuth-based quadruple therapy consisting of PPI plus a standardized three-in-one capsule, bismuth subcitrate potassium, metronidazole, and tetracycline has been recommended [12,13] as the first-line treatment of multidrug-resistant *H. pylori* strains, in particular in areas of high clarithromycin resistance [14]. The increase of the failure rates of the triple therapy in many countries such as in Europe, Korea, Japan, and China, [15] induced the scientific community to evaluate new therapeutical approaches in order to decrease the development of the antibiotic resistance phenomenon. Therefore, the study of the potential anti-*H. pylori* activity of carvacrol and its derivatives could be a starting point useful to assess the therapeutic efficacy of alternative compounds inspired by natural scaffolds [16]. Moreover, carvacrol was shown to exert anti-inflammatory (COX inhibition), antinociceptive and antiulcer activities in vitro and in vivo [17,18], which are useful to reduce damages correlated to *H. pylori* colonization of the human gastric mucosa and associated pathogenesis, whereas the potential of plant essential oils in anticancer treatment has recently obtained many research efforts to overcome drug resistance and multiple side effects. For these reasons, several authors also evaluated the antiproliferative efficacy of carvacrol against human gastric adenocarcinoma (AGS) cells in vitro and in Wistar rats in vivo [19].

Starting from these premises and keeping in mind that the development of drugs from plant secondary metabolites is a topic of the most recent ongoing research and engages large-scale pharmacological screenings of extracts and active compounds, we aimed at designing a large library of carvacrol-based derivatives possessing multiple tuneable functional groups for their chemical modulation to desired properties and assuring the broadest chemical diversity. Indeed, natural product derivatives could shed light on new therapeutic agents against human diseases due to the modulation of the physical-chemical, toxicological and drug-like characteristics of their natural parent compound [20]. This is very important when addressing pathologies such as gastric cancer where a pluralism of causative factors must be faced by a feasible research strategy which can evolve a multi-targeted perspective (one molecule acting on separate targets of the disease). Moreover, this approach can overcome issue related to combination therapy and the possibility of drug-drug interactions.

## 2. Results and Discussion

### 2.1. Chemistry

**Scheme 1:**
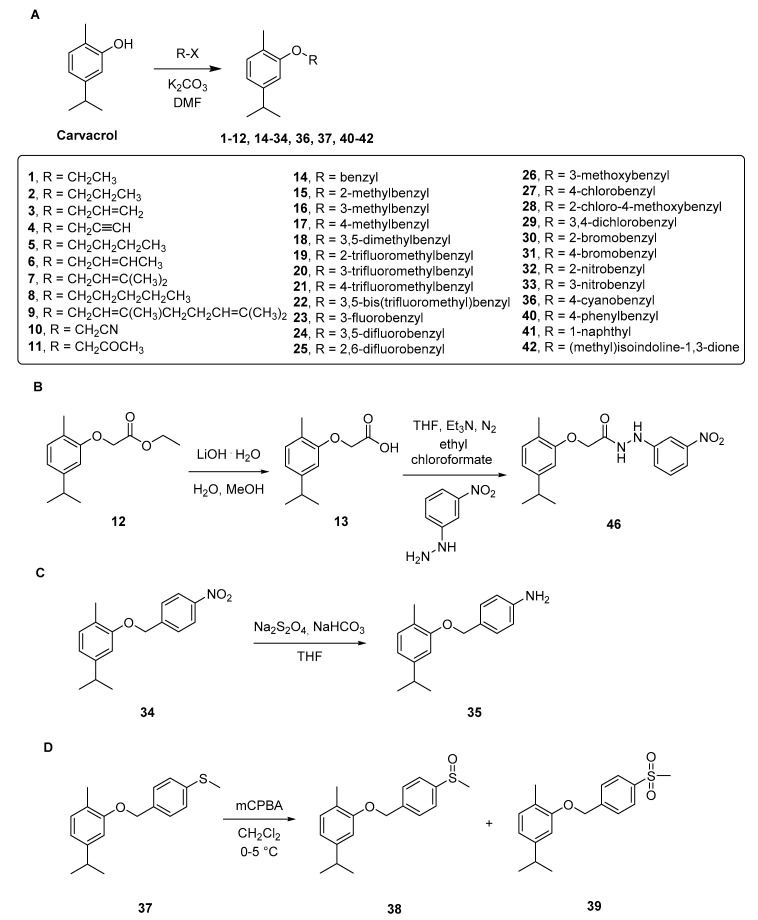
Synthesis of ether compounds **1**–**42** and **46**. (**A**) compounds obtained by direct modification of the hydroxy group of carvacrol; (**B**) alkaline hydrolysis of compound **12** and modification of compound **13** to hydrazido compound **46**; (**C**) nitro reduction to amine compound **35**; (**D**) sulfur oxidation to compounds **38** and **39**.

**Scheme 2:**
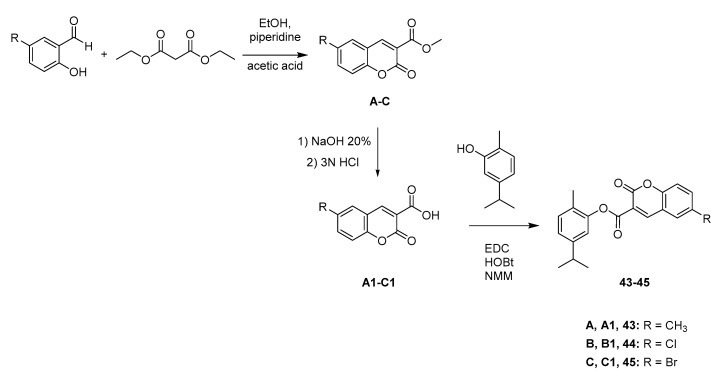
Synthesis of ester compounds **43**–**45** (coumarin-carvacrol hybrids).

For the synthesis of the compounds **1**–**46** we followed the synthetic approach reported in the Scheme 1 and Scheme 2, taking advantage of the hydroxyl portion of carvacrol in order to synthesize ethers and esters with modified hydrophilic/hydrophobic parameters. Ether derivatives **1**–**12**, **14**–**34**, **36**, **37** and **40**–**42** (Scheme 1A) have been synthesized by reacting the carvacrol with the proper bromide; these reactions were performed in *N*,*N*’-dimethylformamide (DMF), in the presence of potassium carbonate (K_2_CO_3_) and under nitrogen (N_2_) atmosphere. Some of the products obtained in this step were further employed for additional structural modifications obtained through ester hydrolysis, nitro reduction and sulfur oxidation.

In particular, compound **12** was involved in a multistep synthesis. Firstly, it was hydrolyzed in mild conditions using lithium hydroxide (LiOH), in a mixture of water and methanol (in the ratio 50:50, *v*:*v*) at room temperature (RT), to provide the carboxylic acid derivative **13**. Secondly, it was reacted with 3-nitrophenylhydrazine in ethanol to achieve the corresponding acetohydrazide **46** (Scheme 1B).

The NO_2_ group, located at the para position of the benzyl moiety of compound **34**, was reduced with sodium dithionite (Na_2_S_2_O_4_), leading to the *p*-NH_2_ derivative **35** (Scheme 1C). Derivative **37**, obtained from the reaction between carvacrol and (4-(bromomethyl)phenyl)(methyl)sulfane, was treated with *m*-chloroperbenzoic acid (mCPBA) in dichloromethane (DCM). This reaction led to the two oxidized forms of sulfane (sulfoxide and sulfone, respectively the compounds **38** and **39**) in the same step, by modulating the amount of the oxidant agent (mCPBA) added [21]. For the synthesis of the ester compounds **43**–**45**, we synthesized the coumarin-3-carboxylic acids at first, which were then used in a condensation reaction with carvacrol (Scheme 2). Through the Knoevenagel condensation between the properly substituted 2-hydroxybenzaldehyde and the diethyl malonate, we obtained the esters **A**–**C** [22]. The removal of the ester function through hydrolysis was performed using 10% NaOH solution and afforded the carboxylic acid derivatives **A1**–**C1**. Finally, coupling of carvacrol with the proper coumarin-3-carboxylic acid, using 1-ethyl-3-(3-dimethylaminopropyl)carbodiimide (EDC) and 1-hydroxybenzotriazole (HOBt) as condensing agents in 4-methylmorpholine (NMM), gave the title compounds **43**–**45**. The selection of this nucleus and its substituents was suggested by the good results obtained in the evaluation of *H. pylori* strains previously published by some of us [23].

The compounds were stable in their solid state at room temperature. The structures were confirmed by spectral studies (^1^H, ^13^C, and ^19^F NMR), whereas the purity of these compounds was confirmed by combustion analysis, X-ray diffraction studies (for compound **34**), TLC parameters and melting point evaluation.

### 2.2. X-ray Diffraction Analysis

**Figure 1:**
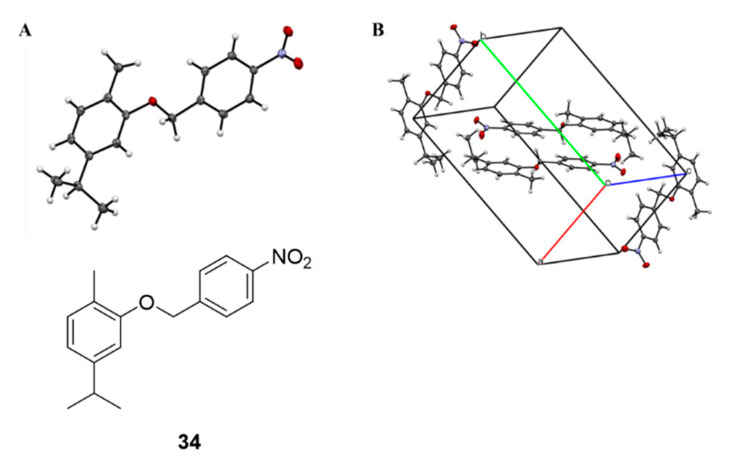
(**A**): ORTEP structure of compound **34**; (**B**) Crystal structure of carvacrol-based compound **34**.

Crystals of compound **34** (Figure 1) were obtained by slow evaporation from an ethyl acetate/*n*-hexane mixture. Information about the crystal data, experimental collection conditions and refinement as well as the structural geometric parameters are available in the Cambridge Crystallographic Data Centre in CIF format and in the Supporting information (Tables S1–S4).

### 2.3. Pan Assay Interference Compounds (PAINS) Evaluation

All designed inhibitors have been analyzed by means of three different theoretical tools, such as ZINC PAINS Pattern Identifier [24], False Positive Remover [25], and FAF-Drug4 [26]. Our compounds were not reported as potential PAINS or covalent inhibitors by none of the considered algorithms.

### 2.4. Biological Assay

After a proper purification and characterization, the compounds were subjected to in vitro biological experiments to assess their inhibitory activity against *H. pylori* growth and AGS cells aiming at discovering the structural requirements to achieve a dual agent.

#### 2.4.1. In Vitro Inhibitory Activity against *H. pylori* Strains

Disposing of all envisioned products, the in vitro inhibitory activity against nine strains of *H. pylori* (one reference strain and eight clinical isolates) characterized by a different antibiotic susceptibility pattern was evaluated and the data reported in Table 1. The susceptibility pattern followed the breakpoints as classified in the international EUCAST (European Committee on Antimicrobial Susceptibility Testing) guidelines for *H. pylori* strains.

The parent compound, carvacrol, displayed MIC and MBC values in the range 16–64 and 32–64 µg/mL, respectively. All the chemical modifications can be grouped into four classes to define robust structure-activity relationships (SARs):

(1) Alkyloxy derivatives **1**–**13**: In general, these derivatives were characterized by an increasing alkyl chain, linear or branched, saturated or unsaturated, functionalized with additional moieties (cyano, ketone, ester, carboxylic acid). None of these modifications led to an improvement of the inhibitory activity with respect to the parent compound. Only derivatives **2** (OPr) and **3** (OBu) slightly presented MIC values comparable to carvacrol against two strains (F34/497 and F40/499), whereas compounds **6** (*O*-crotyl) and **9** (*O*-geranyl) were endowed with inferior MIC and MBC values up to 16 and 8 µg/mL, respectively, toward all the strains;
(2) Benzyloxy derivatives **14**–**40** and **46**: The simplest representative of this class (**14**, *O*-benzyl) had an anti-*H. pylori* activity comparable to carvacrol, whereas other substitutions on the aryl ring such as 3,4-diCl, 2,6-diF, 3-F, 3-OCH_3_, 2-Cl-4-OCH_3_, 2-Br, and 4-Br were detrimental or didn’t produce a strong increment of the antimicrobial activity. Conversely, some substituents, especially in the para position of the aryl ring, such as CF_3_, Ph, CN, NO_2_ and NH_2_ were promising to show improvements. Indeed, CF_3_ could act as a bioisostere of the NO_2_ group and in both series we can highlight the following activity order: *p* > *m* > *o*.
  The presence of a fluorine atom or a trifluoromethyl group into an organic scaffold can lead to changes in the physical, chemical and biological properties, often associated with an increase lipophilicity and electronegativity but a relatively small size, which can favour entry into the cell membranes. The presence of two CF_3_ in compound **22** didn’t synergistically contribute to an improved inhibitory action. As regards sulfur-based compounds (**37**–**39**) we highlighted a better activity with a higher sulfur oxidation state (ArSO_2_CH_3_ > ArSOCH_3_ > ArSCH_3_). Unfortunately, the introduction of bromine atoms in compounds **30** and **31** reduced the inhibitory effect likely due to their low ability to act as H-bond acceptors and their higher atomic radius, both determining a negative steric constrain. Moreover, electron-donating groups were not tolerated.
(3) Bicyclic and heteroaryl derivatives **41** and **42**: The change of the benzyl group into a naphthalene led to a total loss of inhibitory activity, whereas phthalimide can be tolerated;
(4) Coumarin ester derivatives **43**–**45**: These compounds imparted a slight improvement of the anti-*Helicobacter activity* against all the strains with respect to carvacrol. These results were in accordance with those obtained with the same substitution pattern previously published by us [23,27].

Regarding the mechanism of action, it is reasonable to consider these compounds as good bactericidal inhibitors, being the value of MBC/MIC ratio between 1 and 2.

#### 2.4.2. Effects of Carvacrol and Its Derivatives on Cell Viability of AGS Cell Line

As a follow-up study, the selection of the candidates for biological assays was guided by the anti-*Helicobacter pylori* activity (Table 1, compounds highlighted in gray). AGS cells, were incubated for 24 h with the specified molecules or with 0.1% DMSO vehicle (control). Data shown are the means ± SD of three experiments with quintuplicate determinations. Carvacrol showed cytotoxic effects by reducing cell viability of AGS cells (IC_50_ = 300 ± 6.5 μM) in a dose-dependent manner after treatment. Out of 17 carvacrol derivatives, only five (**16**, **21**, **35**, **38** and **39**) demonstrated a dose-dependent inhibitory effect on cell viability inferior to carvacrol. All of them possessed an IC_50_ value higher than the reference drug (5-Fluorouracil, IC_50_ = 82.3 ± 5.6 μM) (Table 2).

More in detail, carvacrol was a medium potency anti-proliferative agent against AGS cells. From the results shown in Table 2, it is possible to highlight that the introduction of alkyl substituents (compounds **6** and **9**) or coumarin rings (compounds **43**–**45**) directly connected to the carvacrol oxygen led to a loss of inhibitory activity. The presence of a benzyl moiety, especially meta or para substituted, exerted some improvements. In particular, 4-CF_3_, 3-CH_3_, 4-SOCH_3_ and 4-SO_2_CH_3_ brought to compounds endowed with a stronger effect with respect to carvacrol. Other clear SAR trends are not observable. These small and easily accessible molecules are promising motifs in the development of dual agents able not only to reduce *H. pylori* growth, but also to counteract the proliferation of AGS cells at higher concentration.

## 3. Materials and Methods

### 3.1. Chemistry

Unless otherwise indicated, all reactions were carried out under a positive pressure of nitrogen in washed and oven-dried glassware. All the solvents and reagents were directly used as supplied by Sigma-Aldrich (Milan, Italy) without further purification. Where mixtures of solvents are specified, the stated ratios are volume:volume. All melting points were measured on a SMP1 melting point apparatus (Stuart^®^, Staffordshire, UK) and are uncorrected (temperatures are reported in °C). Structural analysis consisted of elemental analysis, ^1^H-/^13^C-/^19^F NMR spectra and crystallographic studies. ^1^H and ^13^CNMR spectra were mainly recorded at 300 MHz and 75 MHz (Mercury spectrometer, Varian, Santa Clara, CA, USA), while some compounds were analysed at 400 MHz and 101 MHz on a Bruker spectrometer (Milan, Italy), using CDCl_3_ and DMSO-*d*_6_, as the solvents at room temperature. Conversely, ^19^F spectra were recorded on a Bruker AVANCE 600 spectrometer at 564.7 MHz, using CDCl_3_ as the solvent. All the compounds were studied at the final concentration of ~25 mg/mL. ^1^H and ^13^C chemical shifts are expressed as *δ* units (parts per millions) relative to the solvent signal, whereas ^19^F chemical shifts are expressed as *δ* units relative to an external standard (CF_3_COOH, *δ* −76.55 ppm). ^1^H spectra are described as follows: *δ*_H_ (spectrometer frequency, solvent): chemical shift/ppm (multiplicity, *J*-coupling constant(s) in Hertz (Hz), number of protons, assignment). ^13^C spectra are described as follows: *δ*_C_ (spectrometer frequency, solvent): chemical shift/ppm (assignment) and are fully proton decoupled. ^19^F spectra are described as follows: *δ*_F_ (spectrometer frequency, solvent): chemical shift/ppm (multiplicity, *J*-coupling constant(s) in Hertz, number of fluorine, assignment). Multiplets are abbreviated as follows: br—broad; s—singlet; d—doublet; t—triplet; q—quartet; td—triplet of doublets; m—multiplet. The exchangeable protons (OH, NH_2_) were assessed by the addition of deuterium oxide. The processing and analyses of the NMR data were carried out with MestreNova. Preparative chromatography was carried out employing silica gel (high purity grade, pore size 60 Å, 230–400 mesh particle size). All the purifications and reactions were carried out by thin layer chromatography (TLC) performed on 0.2 mm thick silica gel-aluminium backed plates (60 F254). Spot visualization was performed under short- and long-wavelength (254 and 365 nm, respectively) ultra-violet irradiation. Where given, systematic compound names were generated by ChemBioDraw Ultra 14.0 following IUPAC conventions. Microanalyses were performed with a Perkin-Elmer 260 elemental analyzer (PerkinElmer, Inc., Waltham, MA, USA) for C, H and N and the results were within ±0.4% of the theoretical values. NMR spectra of all new compounds have been reported in the Supplementary Materials.

### 3.2. Synthesis of Carvacrol Derivatives

#### 3.2.1. General Procedure for the Synthesis of Compounds **1**–**12**, **14**–**34**, **36**, **37**, and **40**–**42**

To a stirring solution of carvacrol (1 equiv.) in dry DMF (10 mL) was added freshly ground and anhydrous potassium carbonate (K_2_CO_3_, 1.2 equiv.). The suspension was stirred for 30 min at room temperature; then, the proper (substituted)benzyl, diarylmethyl, heteroarylmethyl or alkyl bromide (1.0 equiv.) was added and the reaction stirred until disappearance of the starting reagents, as detected by TLC. Once the reaction was completed, the mixture was poured into ice-cold water (100 mL) and extracted with dichloromethane (DCM, 3 × 20 mL). The organics were reunited and added with anhydrous sodium sulphate (Na_2_SO_4_) to remove water. The salt was filtered and washed three times with small amounts (5 mL) of dry DCM. The organic phase was evaporated in vacuo to afford the crude extract containing the target molecule that was recovered through column chromatography, employing silica gel (SiO_2_) and proper mixtures of *n*-hexane/ethyl acetate.

#### 3.2.2. Synthesis of Compound **13**

To a stirring solution of ethyl 2-(5-isopropyl-2-methylphenoxy) acetate (**12**, 1.0 equiv.) in 10 mL of methanol was added dropwise lithium hydroxide (1.2 equiv.) dissolved in 10 mL of water. The reaction was stirred at room temperature for 24 h; then, the mixture was concentrated in vacuo to remove methanol and quenched with 3N HCl (15 mL). The precipitate was collected by filtration and washed with *n*-hexane to give the title compound **13**, without further purification requirements.

#### 3.2.3. Synthesis of Compound **46**

To a stirring solution of ethyl 2-(5-isopropyl-2-methylphenoxy)acetic acid (**13**, 1.0 equiv.) in 10 mL of THF was added triethyl amine (3.0 equiv.) and ethyl chloroformate (1.2 equiv.). After 30 min, 3-nitrophenylhydrazine (1.3 equiv.) was added and the reaction stirred at room temperature for 3 h. Once the reaction was completed, the mixture was poured on ice-cold water (100 mL) and the precipitate collected by filtration. Purification through column chromatography (SiO_2_, *n*-hexane:ethyl acetate 2:1) afforded the title compound **46**.

#### 3.2.4. Synthesis of Compound **35**

To a stirring solution of 4-isopropyl-1-methyl-2-((4-nitrobenzyl)oxy)benzene (compound **34**, 1.0 equiv.) in tetrahydrofuran (THF, 15 mL) was added dropwise a freshly prepared solution of sodium dithionite (5.5 equiv.) dissolved in a basic solution made of water (15 mL) and sodium bicarbonate (5.5 equiv.). The reaction was stirred at room temperature until completion (assessed by TLC); then THF was evaporated in vacuo and the aqueous phase extracted with DCM (3 × 20 mL). The organics were reunited, dried over sodium sulphate and filtered to remove the salt. DCM was evaporated in vacuo to give the crude extract, that was purified by column chromatography (SiO_2_, *n*-hexane:ethyl acetate 5:1) to afford the amino derivative **35** as an orange viscous oil.

#### 3.2.5. Synthesis of Compounds **38** and **39**

To a stirring solution of (4-((5-isopropyl-2-methylphenoxy)methyl)phenyl)(methyl) sulfane (**37**, 1 equiv.) in DCM (10 mL) placed on ice/water bath (0–5 °C), was added dropwise a freshly prepared solution of 3-chloroperbenzoic acid (1 equiv.) dissolved in 5 mL of DCM in an ice-bath. The reaction was followed by TLC and after 8 h another aliquot of 3-chloroperbenzoic acid (1 equiv. in 5 mL of DCM) was added and the reaction stirred at room temperature for further 24 h. Once the reaction completion was reached (appearance on TLC of the two spots relative to sulfoxide and sulfone derivatives), the mixture was concentrated in vacuo and the two compounds separated by column chromatography on silica gel (*n*-hexane:ethyl acetate, 5:1) to give the title compounds **38** and **39**.

#### 3.2.6. Synthesis of Intermediates **A**/**A1**-**C**/**C1**

For the synthesis of the coumarin-3-carboxylic acids **A1**–**C1** we used the synthetic procedures previously reported by our group [22]. Briefly, the Knoevenagel cyclization between the proper substituted salicylaldehydes (1 equiv.) and diethyl malonate (1 equiv.) was performed in ethanol (25 mL) with catalytic amounts of piperidine. The reaction was followed by TLC until disappearance of their starting reagents. Once the reaction completed, the mixture was poured into ice-cold water and the solid collected by filtration. The powder was washed with *n*-hexane to obtain the title ester compounds **A**–**C**.

The corresponding ester (**A**, **B** or **C**, 1 equiv.) was then dissolved in ethanol (10 mL) and hydrolyzed by using 20% NaOH solution (25 mL). After reaction completion assessed by means of TLC, the ethanol was evaporated in vacuo. The solution was quenched with 3N HCl (20 mL) leading to precipitation of the coumarin-3-carboxylic acid, that was collected by filtration and washed with *n*-hexane, affording the title compounds **A1**–**C1** without further purification requirements.

#### 3.2.7. Synthesis of Compounds **43**–**45**

To a stirring solution of the proper coumarin-3-carboxylic acid (**A1**–**C1**, 1.0 equiv.) in 4-methylmorpholine (NMM, 10 mL) under nitrogen atmosphere, were added portionwise 1-ethyl-3-(3-dimethylaminopropyl)carbodiimide (EDC, 1.2 equiv.) and 1-hydroxybenzotriazole (HOBt, 1.2 equiv.). After 1 h, carvacrol (1.0 equiv.) was added and the reaction stirred for further 24 h. At the reaction completion (by TLC), the mixture was poured on ice-cold water. The precipitate was collected by filtration and washed with petroleum ether and *n*-hexane to afford title compounds **43**–**45** in good yield and purity.

### 3.3. Characterization Data for Carvacrol Derivatives

2-ethoxy-4-isopropyl-1-methylbenzene (**1**). Colourless oil, 66% yield. ^1^H NMR (400 MHz, CDCl_3_): *δ* 1.29 (d, *J* = 6.9 Hz, 6H, 2 × CH_3_), 1.46 (t, *J* = 7.0 Hz, 3H, CH_2_CH_3_), 2.23 (s, 3H, ArCH_3_), 2.87–2.94 (m, 1H, CH), 4.06–4.11 (m, 2H, OCH_2_CH_3_), 6.74 (s, 1H, Ar), 6.76–6.78 (m, 1H, Ar), 7.10 (d, *J* = 7.5 Hz, 1H, Ar). ^13^C NMR (101 MHz, CDCl_3_): *δ* 15.1 (CH_3_), 15.8 (CH_3_), 24.2 (2 × CH_3_), 34.2 (CH), 63.5 (OCH_2_), 109.6 (Ar), 117.9 (Ar), 124.2 (Ar), 130.4, 147.8 (Ar), 157.1 (Ar). Anal. Calcd for C_12_H_18_O: C, 80.85; H, 10.18. Found: C, 81.12; H, 10.14.

4-isopropyl-1-methyl-2-propoxybenzene (**2**). Colourless oil, 56% yield, mp 117–121 °C. ^1^H NMR (400 MHz, CDCl_3_): *δ* 1.12 (t, *J* = 7.4 Hz, 3H, CH_2_CH_2_CH_3_), 1.31 (d, *J* = 6.9 Hz, 6H, 2 × CH_3_), 1.84–1.93 (m, 2H, CH_2_CH_2_CH_3_), 2.26 (s, 3H, ArCH_3_), 2.89–2.96 (m, 1H, CH), 4.00 (t, *J* = 6.4 Hz 2H, OCH_2_ CH_2_CH_3_), 6.75–6.79 (m, 2H, Ar), 7.11 (d, *J* = 7.5 Hz, 1H, Ar). ^13^C NMR (101 MHz, CDCl_3_): *δ* 10.7 (CH_3_), 15.8 (CH_3_), 22.9 (CH_2_), 24.2 (2 × CH_3_), 34.2 (CH), 69.4 (OCH_2_), 109.5 (Ar), 117.8 (Ar), 124.2 (Ar), 130.4 (Ar), 147.9 (Ar), 157.2 (Ar). Anal. Calcd for C_13_H_20_O: C, 81.20; H, 10.48. Found: C, 81.33; H, 10.51.

2-(allyloxy)-4-isopropyl-1-methylbenzene (**3**). Yellow oil, 70% yield. ^1^H NMR (300 MHz, CDCl_3_): *δ* 1.37 (s, 3H, CH_3_), 1.41 (s, 3H, CH_3_), 2.36 (s, 3H, ArCH_3_), 2.94–3.01 (m, 1H, CH), 4.66-4.69 (m, 2H, CH_2_), 5.37–5.60 (m, 2H, OCH_2_), 6.14–6.27 (m, 1H, =CH), 6.83 (s, 1H, Ar), 6.86-6.89 (m, 1H, Ar), 7.19 (d, *J* = 7.5 Hz, 1H, Ar). ^13^C NMR (75 MHz, CDCl_3_): *δ* 16.0 (CH_3_), 24.3 (2 × CH_3_), 34.3 (CH_3_), 68.8 (OCH_2_), 110.0 (Ar), 116.9 (Ar), 118.3 (=CH_2_), 124.3 (Ar), 130.6 (Ar), 133.9 (=CH), 147.9 (Ar), 156.8 (Ar). Anal. Calcd for C_13_H_18_O: C, 82.06; H, 9.54. Found: C, 81.87; H, 9.58.

4-isopropyl-1-methyl-2-(prop-2-yn-1-yloxy)benzene (**4**). Yellow oil, 74% yield. ^1^H NMR (300 MHz, CDCl_3_): *δ* 1.35 (d, *J* = 6.9 Hz, 6H, 2 × CH_3_), 2.32 (s, 3H, ArCH_3_), 2.58 (t, *J* = 2.4 Hz, 1H, ≡CH), 2.9–3.01 (m, 1H, CH), 4.80 (d, *J* = 2.4 Hz, 1H, OCH_2_), 6.89 (d, *J* = 7.5 Hz, 1H, Ar), 6.93 (s, 1H, Ar), 7.17 (d, *J* = 7.8 Hz, 1H, Ar). ^13^C NMR (75 MHz, CDCl_3_): *δ* 15.9 (CH_3_), 24.2 (2 × CH_3_), 34.2 (CH), 56.01 (OCH_2_), 75.26 (C_sp_), 79.2 (C_sp_H), 110.4 (Ar), 119.2 (Ar), 124.6 (Ar), 130.8 (Ar), 147.9 (Ar), 155.8 (Ar). Anal. Calcd for C_13_H_16_O: C, 82.94; H, 8.57. Found: C, 83.17; H, 8.54.

2-butoxy-4-isopropyl-1-methylbenzene (**5**). Colourless oil, 78% yield. ^1^H NMR (400 MHz, CDCl_3_): *δ* 1.07 (t, *J* = 7.4 Hz, 3H, CH_2_CH_2_CH_2_CH_3_), 1.32 (d, *J* = 6.9 Hz, 6H, 2 × CH_3_), 1.56–1.65 (m, 2H, CH_2_CH_2_CH_2_CH_3_), 1.83–1.90 (m, 2H, CH_2_CH_2_CH_2_CH_3_), 2.27 (s, 3H, ArCH_3_), 2.90–2.97 (m, 1H, CH), 4.05 (t, *J* = 6.3 Hz 2H, OCH_2_ CH_2_CH_3_), 6.77–6.80 (m, 2H, Ar), 7.12 (d, *J* = 7.5 Hz, 1H, 1Ar). ^13^C NMR (101 MHz, CDCl_3_): *δ* 14.0 (CH_3_), 15.9 (CH_3_), 19.5 (CH_2_), 24.2 (2 × CH_3_), 31.6 (CH_2_), 34.2 (CH), 67.6 (OCH_2_), 109.5 (Ar), 117.8 (Ar), 124.2 (Ar), 130.4 (Ar) 147.8 (Ar), 157.2 (Ar). Anal. Calcd for C_14_H_22_O: C, 81.50; H, 10.75. Found: C, 81.63; H, 10.77.

2-(but-2-en-1-yloxy)-4-isopropyl-1-methylbenzene (**6**). Pale yellow oil, 69% yield. ^1^H NMR (300 MHz, CDCl_3_): *δ* 1.55–1.58 (m, 6H, 2 × CH_3_), 2.05 (d, *J* = 4.8 Hz, 3H, CH_3_), 2.54 (s, 3H, ArCH_3_), 3.12–3.19 (m, 1H, CH), 4.76 (d, *J* = 5.1 Hz, 2H, OCH_2_), 6.01–6.18 (m, 2H, 2 × =CH), 7.01 (s, 1H, Ar), 7.03 (d, *J* = 7.8 Hz, 1H, Ar), 7.35 (d, *J* = 7.5 Hz, 1H, Ar). ^13^C NMR (75 MHz, CDCl_3_): *δ* 16.2 (CH_3_), 18.1 (CH_3_), 24.4 (2 × CH_3_), 34.5 (CH), 68.8 (OCH_2_), 110.0 (Ar), 118.3 (Ar), 124.4 (Ar), 127.1 (=CH), 129.4 (=CH), 130.7 Ar), 147.9 (Ar), 157.2 (Ar). Mixture of *E*/*Z* isomers with 5.2:1 ratio. For sake of clarity, we reported only the signals related to the major isomer. Anal. Calcd for C_14_H_20_O: C, 82.30; H, 9.87. Found: C, 82.33; H, 9.88.

4-isopropyl-1-methyl-2-((3-methylbut-2-en-1-yl)oxy)benzene (**7**). Yellow oil, 71% yield. ^1^H NMR (300 MHz, CDCl_3_): *δ* 1.29–1.32 (m, 6H, 2 × CH_3_), 1.81–1.85 (m, 6H, 2 × CH_3_), 2.26 (s, 3H, ArCH_3_), 2.87–2.96 (m, 1H, CH), 4.95 (d, *J* = 6.6 Hz, 2H, OCH_2_), 5.54–5.58 (m, 1H, =CH), 6.77 (s, 1H, Ar), 6.79–6.80 (m, 1H, Ar), 7.11 (d, *J* = 7.5 Hz, 1H, Ar). ^13^C NMR (75 MHz, CDCl_3_): *δ* 16.0 (CH_3_), 18.3 (CH_3_), 24.2 (2 × CH_3_), 25.9 (CH_3_), 34.2 (CH), 65.0 (OCH_2_), 110.0 (Ar), 118.0 (-CH=), 120.5 (Ar), 124.4 (Ar), 130.4 (Ar), 137.0 (=C), 147.8 (Ar), 157.0 (Ar). Anal. Calcd for C_15_H_22_O: C, 82.52; H, 10.16. Found: C, 82.61; H, 10.13.

4-isopropyl-1-methyl-2-(pentyloxy)benzene (**8**). Colourless oil, 78% yield. ^1^H NMR (400 MHz, CDCl_3_): *δ* 1.01–1.06 (m, 3H, CH_2_CH_2_CH_2_CH_2_CH_3_), 1.35 (d, *J* = 6.0 Hz, 6H, 2 × CH_3_), 1.45–1.61 (m, 4H, 2 × CH_2_, CH_2_CH_2_CH_2_CH_2_CH_3_), 1.86–1.97 (m, 2H, CH_2_CH_2_CH_2_CH_2_CH_3_), 2.29 (s, 3H, ArCH_3_), 2.92–2.99 (m, 1H, CH), 4.06 (t, *J* = 6.4 Hz 2H, OCH_2_CH_2_CH_2_CH_2_CH_3_), 6.78–6.82 (m, 2H, Ar), 7.14 (d, *J* = 7.5 Hz, 1H, 1Ar). ^13^C NMR (101 MHz, CDCl_3_): *δ* 14.1 (CH_3_), 15.9 (CH_3_), 22.6 (CH_2_), 24.2 (2 × CH_3_), 28.5 (CH_2_), 29.2 (CH_2_), 34.2 (CH), 67.9(OCH_2_), 109.5 (Ar), 117.8 (Ar), 124.2 (Ar), 130.4 (Ar) 147.9 (Ar), 157.3 (Ar). Anal. Calcd for C_15_H_24_O: C, 81.76; H, 10.98. Found: C, 81.88; H, 11.01.

2-((3,7-dimethylocta-2,6-dien-1-yl)oxy)-4-isopropyl-1-methylbenzene (**9**). Yellow oil, 74% yield. ^1^H NMR (300 MHz, CDCl_3_): *δ* 1.29–1.31 (m, 6H, 2 × CH_3_), 1.67 (s, 3H, CH_3_), 1.74 (s, 3H, CH_3_), 1.80 (s, 3H, CH_3_), 2.10–2.20 (m, 4H, 2 × CH_2_), 2.26 (s, 3H, ArCH_3_), 2.89–2.94 (m, 1H, CH), 4.62 (d, *J* = 6.3 Hz, 2H, OCH_2_), 5.16–5.17 (m, 1H, =CH), 5.55–5.56 (m, 1H, =CH), 6.77 (s, 1H, Ar), 6.77–6.79 (m, 1H, Ar), 7.10 (d, *J* = 7.5 Hz, 1H, Ar). ^13^C NMR (75 MHz, CDCl_3_): *δ* 16.0 (CH_3_), 16.7 (CH_3_), 17.7 (CH_3_), 24.2 (2 × CH_3_), 25.7 (CH_3_), 26.4 (CH_2_), 34.2 (CH), 39.6 (CH_2_), 65.0 (OCH_2_), 109.9 (Ar), 118.0 (Ar), 120.4 (=CH), 123.9 (Ar), 124.3 (=CH), 130.4 (Ar), 131.7 (=C), 140.1 (=C), 147.7 (Ar), 157.0 (Ar). Mixture of *E*/*Z* isomers with 2:1 ratio. For sake of clarity, we reported only the signals related to the major isomer. Anal. Calcd for C_20_H_30_O: C, 83.86; H, 10.56. Found: C, 84.01; H, 10.51.

2-(5-isopropyl-2-methylphenoxy)acetonitrile (**10**). Colourless oil, 79% yield. ^1^H NMR (300 MHz, CDCl_3_): *δ* 1.30 (d, *J* = 7.2 Hz, 6H, 2 × CH_3_), 2.33 (s, 3H, ArCH_3_), 2.97–3.06 (m, 1H, CH), 4.79 (s, 2H, OCH_2_), 6.89 (s, 1H, Ar), 6.97–7.00 (m, 1H, Ar), 7.21 (d, *J* = 7.8 Hz, 1H, Ar). ^13^C NMR (75 MHz, CDCl_3_): *δ* 15.7 (CH_3_), 24.1 (2 × CH_3_), 34.1 (CH), 53.9 (OCH_2_), 110.5 (Ar), 115.8 (CN), 120.8 (Ar), 124.9 (Ar), 131.3 (Ar), 148.4 (Ar), 154.9 (Ar). Anal. Calcd for C_12_H_15_NO: C, 76.16; H, 7.99; N, 7.40. Found: C, 76.27; H, 8.00; N, 7.38.

1-(5-isopropyl-2-methylphenoxy)propan-2-one (**11**). Yellow oil, 80% yield. ^1^H NMR (300 MHz, CDCl_3_): *δ* 1.27 (d, *J* = 6.9 Hz, 6H, 2 × CH_3_), 2.31 (s, 3H, CH_3_), 2.35 (s, 3H, CH_3_), 2.84–2.94 (m, 1H, CH), 4.55 (s, 2H, OCH_2_), 6.60 (s, 1H, Ar), 6.82–6.84 (m, 1H, Ar), 7.13 (d, *J* = 7.8 Hz, 1H, Ar). ^13^C NMR (75 MHz, CDCl_3_): *δ* 15.9 (CH_3_), 24.1 (2 × CH_3_), 26.7 (CH_3_), 34.1 (CH), 73.2 (OCH_2_), 109.3 (Ar), 119.1 (Ar), 124.1 (Ar), 130.9 (Ar), 148.1 (Ar), 155.9 (Ar), 206.5 (C=O). Anal. Calcd for C_13_H_18_O_2_: C, 75.69; H, 8.80. Found: C, 75.84; H, 8.77.

Ethyl 2-(5-isopropyl-2-methylphenoxy)acetate (**12**). Yellow oil, 77% yield. ^1^H NMR (300 MHz, CDCl_3_): *δ* 1.28–1.33 (m, 3H, CH_2_CH_3_), 1.34 (s, 3H, CH_3_), 1.36 (s, 3H, CH_3_), 2.34 (s, 3H, ArCH_3_), 2.86–2.93 (m, 1H, CH), 4.26–4.33 (m, 2H, CH_2_CH_3_), 4.68 (s, 2H, OCH_2_), 6.67 (s, 1H, Ar), 6.83 (d, *J* = 7.8 Hz, 1H, Ar), 7.10 (d, *J* = 7.8 Hz, 1H, Ar). ^13^C NMR (75 MHz, CDCl_3_): *δ* 14.2 (CH_3_), 15.8 (CH_3_), 24.1 (2 × CH_3_), 34.1 (CH_3_), 61.0 (CH_2_CH_3_), 65.7 (OCH_2_), 109.7 (Ar), 119.2 (Ar), 124.5 (Ar), 130.8 (Ar), 147.7 (Ar), 156.2 (Ar), 169.1 (Ar). Anal. Calcd for C_14_H_20_O_3_: C, 71.16; H, 8.53. Found: C, 71.22; H, 8.51.

2-(5-isopropyl-2-methylphenoxy)acetic acid (**13**). Viscous white oil, 91% yield. ^1^H NMR (300 MHz, CDCl_3_): *δ* 1.23 (d, *J* = 6.9 Hz, 6H, 2 × CH_3_), 2.26 (s, 3H, ArCH_3_), 2.85–2.95 (m, 1H, CH), 4.70 (s, 2H, OCH_2_), 6.61 (s, 1H, Ar), 6.81–6.83 (m, 1H, Ar), 7.08–7.11 (m, 1H, Ar). ^13^C NMR (75 MHz, CDCl_3_): *δ* 15.7 (CH_3_), 24.0 (2 × CH_3_), 34.0 (CH_3_), 65.3 (OCH_2_), 110.0 (Ar), 119.8 (Ar), 124.5 (Ar), 131.0 (Ar), 148.1 (Ar), 155.5 (Ar). Anal. Calcd for C_12_H_16_O_3_: C, 69.21; H, 7.74. Found: C, 69.11; H, 7.71.

2-(benzyloxy)-4-isopropyl-1-methylbenzene (**14**). Yellow oil, 88% yield. ^1^H NMR (300 MHz, CDCl_3_): *δ* 1.54 (m, 6H, 2 × CH_3_), 2.57 (s, 3H, ArCH_3_), 3.11–3.20 (m, 1H, CH), 5.34 (s, 2H, OCH_2_), 7.04–7.07 (m, 2H, Ar), 7.37 (d, *J* = 7.8 Hz, 1H, Ar), 7.54–7.67 (m, 3H, Ar), 7.73 (d, *J* = 6.9 Hz, 2H, Ar). ^13^C NMR (75 MHz, CDCl_3_): *δ* 16.3 (CH_3_), 24.4 (2 × CH_3_), 34.4 (CH), 70.1 (OCH_2_), 110.2 (Ar), 118.6 (Ar), 124.6 (Ar), 127.5 (2 × Ar), 128.0 (Ar), 128.7 (2 × Ar), 130.8 (Ar), 137.9 (Ar), 148.1 (Ar), 157.1 (Ar). Anal. Calcd for C_17_H_20_O: C, 84.96; H, 8.39. Found: C, 85.01; H, 8.42.

4-isopropyl-1-methyl-2-((2-methylbenzyl)oxy)benzene (**15**). Yellow oil, 86% yield. ^1^H NMR (300 MHz, CDCl_3_): *δ* 1.40 (d, *J* = 6.9 Hz, 6H, 2 × CH_3_), 2.37 (s, 3H, ArCH_3_), 2.52 (s, 3H, ArCH_3_), 2.96–3.10 (m, 1H, CH), 5.16 (s, 2H, OCH_2_), 6.90–6.95 (m, 2H, Ar), 7.22 (d, *J* = 7.5 Hz, 1H, Ar), 7.35–7.37 (m, 3H, Ar), 7.50–7.52 (m, 1H, Ar). ^13^C NMR (75 MHz, CDCl_3_): *δ* 16.1 (CH_3_), 19.1 (CH_3_), 24.3 (2 × CH_3_), 34.3 (CH), 68.5 (OCH_2_), 109.9 (Ar), 118.3 (Ar), 124.5 (Ar), 126.1 (Ar), 128.1 (Ar), 128.5 (Ar), 130.4 (Ar), 130.7 (Ar), 135.5 (Ar), 136.7 (Ar), 148.0 (Ar). Anal. Calcd for C_18_H_22_O: C, 84.99; H, 8.72. Found: C, 85.17; H, 8.70.

4-isopropyl-1-methyl-2-((3-methylbenzyl)oxy)benzene (**16**). Pale yellow oil, 85% yield. ^1^H NMR (300 MHz, CDCl_3_): *δ* 1.58 (d, *J* = 6.9 Hz, 6H, 2 × CH_3_) 2.60 (s, 3H, ArCH_3_), 2.67 (s, 3H, ArCH_3_), 3.16–3.21 (m, 1H, CH), 5.33 (s, 2H, OCH_2_), 7.07–7.10 (m, 2H, Ar), 7.38–7.43 (m, 2H, Ar), 7.56–7.58 (m, 3H, Ar). ^13^C NMR (75 MHz, CDCl_3_): *δ* 16.4 (CH_3_), 21.7 (CH_3_), 24.5 (2 × CH_3_), 34.5 (CH), 70.2 (OCH_2_), 110.2 (Ar), 118.6 (Ar), 124.7 (Ar), 128.3 (Ar), 128.7 (Ar), 128.8 (Ar), 130.8 (Ar), 137.9 (Ar), 138.3 (Ar), 148.1 (Ar), 157.2 (Ar). Anal. Calcd for C_18_H_22_O: C, 84.99; H, 8.72. Found: C, 85.11; H, 8.73.

4-isopropyl-1-methyl-2-((4-methylbenzyl)oxy)benzene (**17**). Yellow oil, 73% yield. ^1^H NMR (300 MHz, CDCl_3_): *δ* 1.61 (d, *J* = 6.9 Hz, 6H, 2 × CH_3_), 2.61 (s, 3H, ArCH_3_), 2.67 (s, 3H, ArCH_3_), 3.18–3.21 (m, 1H, CH), 5.35 (s, 2H, OCH_2_), 7.09–7.13 (m, 2H, Ar), 7.42 (d, *J* = 6.9 Hz, 2H, Ar), 7.50 (d, *J* = 8.4 Hz, 2H, Ar), 7.68 (d, *J* = 7.5 Hz, 2H, Ar). ^13^C NMR (75 MHz, CDCl_3_): *δ* 16.4 (CH_3_), 21.5 (CH_3_), 24.6 (2 × CH_3_), 34.5 (CH), 70.0 (OCH_2_), 110.1 (Ar), 118.5 (Ar), 124.6 (Ar), 127.7 (2 × Ar), 129.5 (2 × Ar), 130.9 (Ar), 134.9 (Ar), 137.6 (Ar), 148.1 (Ar), 157.3 (Ar). Anal. Calcd for C_18_H_22_O: C, 84.99; H, 8.72. Found: C, 84.88; H, 8.69.

2-((3,5-dimethylbenzyl)oxy)-4-isopropyl-1-methylbenzene (**18**). Yellow oil, 63% yield. ^1^H NMR (400 MHz, CDCl_3_): *δ* 1.27–1.30 (m, 6H, 2 × CH_3_), 2.29–2.30 (m, 3H, ArCH_3_), 2.38 (bs, 6H, 2 × ArCH_3_), 2.89–2.93 (m, 1H, CH), 5.05 (s, 2H, OCH_2_), 6.79–6.83 (m, 2H, 2 × Ar), 7.00 (s, 1H, Ar), 7.13 (bs, 3H, 3 × Ar). ^13^C NMR (101 MHz, CDCl_3_): *δ* 16.0 (CH_3_), 21.4 (2 × CH_3_), 24.2 (2 × CH_3_), 34.1 (CH), 70.1 (OCH_2_), 110.2 (Ar), 118.3 (Ar), 124.5 (Ar), 125.2 (2 × Ar), 129.4 (2 × Ar), 130.5 (Ar), 137.5 (Ar), 138.0 (Ar), 147.9 (Ar), 157.0 (Ar). Anal. Calcd for C_19_H_24_O: C, 85.03; H, 9.01. Found: C, 85.23; H, 9.05.

4-isopropyl-1-methyl-2-((2-(trifluoromethyl)benzyl)oxy)benzene (**19**). Colourless oil, 84% yield. ^1^H NMR (300 MHz, CDCl_3_): *δ* 1.60 (d, *J* = 6.3 Hz, 6H, 2 × CH_3_), 2.70 (s, 3H, ArCH_3_), 3.17–3.26 (m, 1H, CH), 5.67 (s, 2H, OCH_2_), 7.13–7.17 (m, 2H, Ar), 7.46 (d, *J* = 7.2, 1H, Ar), 7.62–7.67 (m, 1H, Ar), 7.81–7.86 (m, 1H, Ar), 8.01 (d, *J* = 8.1 Hz, 1H, Ar), 8.17 (d, *J* = 8.1 Hz, 1H, Ar). ^13^C NMR (75 MHz, CDCl_3_): *δ* 16.3 (CH_3_), 24.3 (2 × CH_3_), 34.4 (CH), 66.1 (OCH_2_), 110.2 (Ar), 119.0 (Ar), 123.3 (CF_3_), 124.5 (Ar), 126.0 (Ar), 126.1 (Ar), 127.7 (Ar), 128.6 (Ar), 131.0 (Ar), 132.4 (Ar), 136.7 (Ar), 148.3 (Ar), 156.7 (Ar). ^19^F NMR (564.7 MHz, CDCl_3_): *δ* −58.63 (s, 3F, ArCF_3_). Anal. Calcd for C_18_H_19_F_3_O: C, 70.12; H, 6.21. Found: C, 69.98; H, 6.20.

4-isopropyl-1-methyl-2-((3-(trifluoromethyl)benzyl)oxy)benzene (**20**). Colourless oil, 78% yield. ^1^H NMR (300 MHz, CDCl_3_): *δ* 1.58 (d, *J* = 6.9 Hz, 6H, 2 × CH_3_), 2.59 (s, 3H, ArCH_3_), 3.14–3.23 (m, 1H, CH), 5.36 (s, 2H, OCH_2_), 7.09 (s, 1H, Ar), 7.11–7.12 (m, 1H, Ar), 7.40 (d, *J* = 7.5 Hz, 1H, Ar), 7.69–7.74 (m, 1H, Ar), 7.84 (d, *J* =7.5 Hz, 1H, Ar), 7.90 (d, *J* = 7.5 Hz, 1H Ar), 8.06 (s, 1H, Ar). ^13^C NMR (75 MHz, CDCl_3_): *δ* 16.1 (CH_3_), 24.3 (2 × CH_3_), 34.4 (CH), 69.2 (OCH_2_), 110.0 (Ar), 119.0 (Ar), 124.0 (Ar), 124.0 (Ar), 124.1 (Ar), 124.6 (CF_3_), 124.7 (Ar), 129.2 (Ar), 130.6 (Ar), 131.0 (Ar), 139.0 (Ar), 148.3 (Ar), 156.8 (Ar). ^19^F NMR (564.7 MHz, CDCl_3_): *δ* –60.92 (s, 3F, ArCF_3_). Anal. Calcd for C_18_H_19_F_3_O: C, 70.12; H, 6.21. Found: C, 70.35; H, 6.22.

4-isopropyl-1-methyl-2-((4-(trifluoromethyl)benzyl)oxy)benzene (**21**). Viscous colourless oil, 84% yield. ^1^H NMR (300 MHz, CDCl_3_): *δ* 1.46 (d, *J* = 7.2 Hz, 6H, 2 × CH_3_), 2.49 (s, 3H, ArCH_3_), 3.00–3.12 (m, 1H, CH), 5.30 (s, 2H, OCH_2_), 6.96 (s, 1H, Ar), 6.98–7.01 (m, 1H, Ar), 7.30 (d, *J* = 7.5 Hz, 1H, Ar), 7.74 (d, *J* = 8.4 Hz, 2H, Ar), 7.82 (d, *J* = 8.4 Hz, 2H, Ar). ^13^C NMR (75 MHz, CDCl_3_): *δ* 16.1 (CH_3_)_,_ 24.2 (2 × CH_3_), 34.3 (CH), 69.0 (OCH_2_), 110.0 (Ar), 118.9 (Ar), 124.5 (Ar), 125.5 (2 × Ar), 125.6 (Ar), 127.3 (Ar), 130.3 (CF_3_), 130.9 (Ar), 141.9 (Ar), 148.2 (Ar), 156.6 (Ar). ^19^F NMR (564.7 MHz, CDCl_3_): *δ* −60.77 (s, 3F, ArCF_3_). Anal. Calcd for C_18_H_19_F_3_O: C, 70.12; H, 6.21. Found: C, 70.17; H, 6.19.

2-((3,5-bis(trifluoromethyl)benzyl)oxy)-4-isopropyl-1-methylbenzene (**22**). Yellow oil, 80% yield. ^1^H NMR (400 MHz, CDCl_3_): *δ* 1.26–1.28 (d, *J* =6.8 Hz, 6H, 2 × CH_3_), 2.30 (s, 3H, CH_3_), 2.87–2.94 (m, 1H, CH), 5.21 (s, 2H, CH_2_), 6.78 (s, 1H, Ar), 6.83–6.85 (d, *J* = 7.6, 1H, Ar), 7.14–7.16 (d, *J* = 7.6, 1H, Ar), 7.88 (s, 1H, Ar), 7.97 (s, 2H, 2 × Ar). ^13^C NMR (101 MHz, CDCl_3_): *δ* 15.9 (CH_3_), 24.1 (2 × CH_3_), 34.1 (CH), 68.5 (OCH_2_), 109.9 (Ar), 119.3 (Ar), 121.7 (Ar), 121.9 (Ar), 124.4 (Ar), 124.6 (Ar), 127.1 (Ar), 130.9 (2 × Ar), 132.0 (2 × CF_3_), 140.3 (Ar), 148.1 (Ar), 156.1 (Ar). ^19^F (564.7 MHz, CDCl_3_): *δ* –66.85 (s, 6F, 2 × ArCF_3_). Anal. Calcd for C_19_H_18_F_6_O: C, 60.64; H, 4.82. Found: C, 60.80; H, 4.81.

2-((3-fluorobenzyl)oxy)-4-isopropyl-1-methylbenzene (**23**). Colourless oil, 82% yield. ^1^H NMR (300 MHz, CDCl_3_): *δ* 1.59–1.63 (m, 6H, 2 × CH_3_), 2.63 (s, 3H, ArCH_3_), 3.17–3.24 (m, 1H, CH), 5.34 (s, 2H, OCH_2_), 7.10–7.14 (m, 2H, Ar), 7.26–7.31 (m, 1H, Ar), 7.41–7.45 (m, 1H, Ar), 7.50–7.63 (m, 3H, Ar). ^13^C NMR (75 MHz, CDCl_3_): *δ* 16.3 (CH_3_), 24.4 (2 × CH_3_), 34.5 (CH), 69.2 (OCH_2_), 110.1 (Ar), 114.2 (Ar), 114.8 (Ar), 118.9 (Ar), 122.7 (Ar), 124.6 (Ar), 130.3 (Ar), 131.0 (Ar), 140.7 (Ar), 148.2 (Ar), 156.9 (Ar) 163.4 (d, ^C-F^*J* = 244.9 Hz, C-F). ^19^F NMR (564.7 MHz, CDCl_3_): *δ* −111.26 (td, ^F-H^*J* = 9.0 Hz, 6.0 Hz, 1F, ArF). Anal. Calcd for C_17_H_19_FO: C, 79.04; H, 7.41. Found: C, 78.91; H, 7.39.

2-((3,5-difluorobenzyl)oxy)-4-isopropyl-1-methylbenzene (**24**). Colourless oil, 80% yield. ^1^H NMR (400 MHz, CDCl_3_): *δ* 1.27–1.29 (d, *J* = 6.8 Hz, 6H, 2 × 3CH_3_), 2.31 (s, 3H, CH_3_), 2.87–2.94 (m, 1H, CH), 5.10 (s, 2H, CH_2_), 6.75 (s, 1H, Ar), 6.76–6.84 (m, 2H, 2 × Ar), 7.01–7.06 (m, 2H, Ar), 7.13–7.15 (d, *J* = 7.6 Hz, 1H, Ar). ^13^C NMR (101 MHz, CDCl_3_): *δ* 16.0 (CH_3_), 24.1 (2 × CH_3_), 34.1 (CH), 68.6 (OCH_2_), 102.9 (Ar), 109.6 (Ar), 118.9 (Ar), 124.4 (Ar), 130.7 (Ar), 141.8 (Ar), 148.0 (Ar), 156.3 (Ar), 161.9 (Ar), 164.4 (Ar). ^19^F (564.7 MHz, CDCl_3_): *δ* –113.48 (m, 2F, ArF). Anal. Calcd for C_17_H_18_F_2_O: C, 73.89; H, 6.57. Found: C, 73.94; H, 6.58.

1,3-difluoro-2-((5-isopropyl-2-methylphenoxy)methyl)benzene (**25**). Colourless oil, 89% yield. ^1^H NMR (300 MHz, CDCl_3_): *δ* 1.65 (d, *J* = 6.9 Hz, 6H, 2 × CH_3_), 2.56 (s, 3H, ArCH_3_), 3.20–3.29 (m, 1H, CH), 5.50 (s, 2H, OCH_2_), 7.13–7.19 (m, 3H, Ar), 7.30 (s, 1H, Ar), 7.41 (d, *J* = 7.5 Hz, 1H, Ar), 7.44–7.54 (m, 1H, Ar). ^13^C NMR (75 MHz, CDCl_3_): *δ* 16.0 (CH_3_), 24.4 (2 × CH_3_), 34.5 (CH), 58.4 (OCH_2_), 110.7 (Ar), 111.5 (Ar), 111.7 (Ar), 113.6 (Ar), 119.3 (Ar), 125.1 (Ar), 130.7 (Ar), 131.0 (Ar), 148.2 (Ar), 157.0 (Ar), 162.3 (d, ^C-F^*J* = 248.3 Hz, C-F), 162.3 (d, ^C-F^*J* = 249.5 Hz, C-F). ^19^F NMR (564.7 MHz, CDCl_3_): *δ* −112.80 (t, ^F-H^*J* = 6.6 Hz, 2F, ArF). Anal. Calcd for C_17_H_18_F_2_O: C, 73.89; H, 6.57. Found: C, 74.00; H, 6.58.

4-isopropyl-2-((3-methoxybenzyl)oxy)-1-methylbenzene (**26**). Colourless oil, 80% yield. ^1^H NMR (400 MHz, CDCl_3_): *δ* 1.26–1.29 (m, 6H, 2 × CH_3_), 2.30 (s, 3H, ArCH_3_), 2.87–2.92 (m, 1H, CH), 3.86 (s, 3H, OCH_3_), 5.10 (s, 2H, OCH_2_), 6.81 (bs, 2H, 2 × Ar), 6.89–6.91 (d, *J* = 8.4 Hz, 1H, Ar), 7.07 (bs, 2H, 2 × Ar), 7.12–7.14 (d, *J* = 7.6 Hz, 1H, Ar), 7.32–7.36 (t, *J* = 8.2 Hz, 1H, Ar). ^13^C NMR (101 MHz, CDCl_3_): *δ* 16.0 (CH_3_), 24.1 (2 × CH_3_), 34.1 (CH), 55.2 (OCH_3_), 69.8 (OCH_2_), 110.0 (Ar), 112.7 (Ar), 113.2 (Ar), 118.4 (Ar), 119.4 (Ar), 124.4 (Ar), 129.5 (Ar), 130.5 (Ar), 139.3 (Ar), 147.9 (Ar), 156.8 (Ar), 159.8 (Ar). Anal. Calcd for C_18_H_22_O_2_: C, 79.96; H, 8.20. Found: C, 80.11; H, 8.5.

2-((4-chlorobenzyl)oxy)-4-isopropyl-1-methylbenzene (**27**). Pale yellow oil, 79% yield. ^1^H NMR (300 MHz, CDCl_3_): *δ* 1.52 (d, *J* = 6.9 Hz, 6H, 2 × CH_3_), 2.52 (s, 3H, ArCH_3_), 3.11–3.20 (m, 1H, CH), 5.24 (s, 2H, OCH_2_), 7.01–7.04 (m, 2H, Ar), 7.34 (d, *J* = 7.8 Hz, 1H, Ar), 7.54–7.61 (m, 4H, Ar). ^13^C NMR (75 MHz, CDCl_3_): *δ* 16.3 (CH_3_), 24.4 (2 × CH_3_), 34.4 (CH), 69.2 (OCH_2_), 110.2 (Ar), 118.8 (Ar), 124.5 (Ar), 128.7 (2 × Ar), 128.8 (2 × Ar), 130.9 (Ar), 133.7 (Ar), 136.4 (Ar), 148.1 (Ar), 156.9 (Ar). Anal. Calcd for C_17_H_19_ClO: C, 74.31; H, 6.97. Found: C, 74.43; H, 7.00.

2-chloro-1-((5-isopropyl-2-methylphenoxy)methyl)-4-methoxybenzene (**28**). Colourless oil, 82% yield. ^1^H NMR (400 MHz, CDCl_3_): *δ* 1.26–1.28 (d, *J* = 7.2 Hz, 6H, 2 × CH_3_), 2.28 (s, 3H, CH_3_), 2.87–2.92 (m, 1H, CH), 3.84 (s, 3H, OCH_3_), 5.13 (s, 2H, CH_2_), 6.79–6.81 (m, 2H, 2 × Ar), 6.86–6.89 (m, 1H, Ar), 6.99–7.00 (d, *J* = 2.4 Hz, 1H, Ar), 7.10–7.12 (d, *J* = 7.6 Hz, 1H, Ar), 7.50–7.52 (d, *J* = 8.4 Hz, 2H, 2 × Ar). ^13^C NMR (101 MHz, CDCl_3_): *δ* 16.0 (CH_3_), 24.1 (2 × CH_3_), 34.1 (CH), 55.6 (OCH_3_), 66.9 (OCH_2_), 110.2 (Ar), 112.9 (Ar), 114.8 (Ar), 118.5 (Ar), 124.4 (Ar), 127.3 (Ar), 129.9 (Ar), 130.5 (Ar), 133.5 (Ar), 148.0 (Ar), 156.6 (Ar), 159.7 (Ar). Anal. Calcd for C_18_H_21_ClO_2_: C, 70.93; H, 6.94. Found: C, 71.10; H, 6.92.

1,2-dichloro-4-((5-isopropyl-2-methylphenoxy)methyl)benzene (**29**). Colourless oil, 92% yield. ^1^H NMR (300 MHz, CDCl_3_): *δ* 1.59 (d, *J* = 6.9 Hz, 6H, 2 × CH_3_), 2.58 (s, 3H, ArCH_3_), 3.16–3.21 (m, 1H, CH), 5.22 (s, 2H, OCH_2_), 7.05 (s, 1H, Ar), 7.10 (d, *J* = 7.5 Hz, 1H, Ar), 7.39 (d, *J* = 7.5 Hz, 1H, Ar), 7.48–7.52 (m, 1H, Ar), 7.64 (d, *J* = 8.1 Hz, 1H, Ar), 7.79–7.80 (m, 1H, Ar). ^13^C NMR (75 MHz, CDCl_3_): *δ* 16.4 (CH_3_), 24.5 (2 × CH_3_), 34.5 (CH), 68.5 (OCH_2_), 110.0 (Ar), 119.1 (Ar), 124.5 (Ar), 126.6 (Ar), 129.2 (Ar), 130.7 (Ar), 131.1 (Ar), 131.8 (Ar), 132.8 (Ar), 138.3 (Ar), 148.2 (Ar), 156.7 (Ar). Anal. Calcd for C_17_H_18_Cl_2_O: C, 66.03; H, 5.87. Found: C, 65.94; H, 5.88.

2-((2-bromobenzyl)oxy)-4-isopropyl-1-methylbenzene (**30**). White powder, 61% yield, mp 51–52 °C. ^1^H NMR (300 MHz, CDCl_3_): *δ* 1.27 (d, *J* = 6.9 Hz, 6H, 2 × CH_3_), 2.32 (s, 3H, ArCH_3_), 2.83–2.94 (m, 1H, CH), 5.16 (s, 2H, OCH_2_), 6.79–6.82 (m, 2H, Ar), 7.13 (d, *J* = 7.8 Hz, 1H, Ar), 7.16–7.23 (m, 1H, Ar), 7.34–7.40 (t, 1H, Ar), 7.60–7.65 (m, 2H, Ar). ^13^C NMR (75 MHz, CDCl_3_): *δ* 16.1 (CH_3_), 24.2 (2 × CH_3_), 34.1 (CH), 69.2 (OCH_2_), 110.1 (Ar), 118.5 (Ar), 122.1 (Ar), 124.4 (Ar), 127.6 (Ar), 128.7 (Ar), 129.0 (Ar), 130.6 (Ar), 132.5 (Ar), 136.9 (Ar), 148.0 (Ar), 156.4 (Ar). Anal. Calcd for C_17_H_19_BrO: C, 63.96; H, 6.00. Found: C, 63.85; H, 5.99.

2-((4-bromobenzyl)oxy)-4-isopropyl-1-methylbenzene (**31**). Colourless oil, 66% yield. ^1^H NMR (300 MHz, CDCl_3_): *δ* 1.55 (d, *J* = 6.9 Hz, 6H, 2 × CH_3_), 2.56 (s, 3H, ArCH_3_), 3.12–3.18 (m, 1H, CH), 5.25 (s, 2H, OCH_2_), 7.04–7.09 (m, 2H, Ar), 7.38 (d, *J* = 7.2 Hz, 1H, Ar), 7.55 (d, *J* = 8.1 Hz, 2H, Ar), 7.71–7.75 (m, 2H, Ar). ^13^C NMR (75 MHz, CDCl_3_): *δ* 16.4 (CH_3_), 21.5 (CH_3_), 24.5 (2 × CH_3_), 34.5 (CH), 69.3 (OCH_2_), 110.0 (Ar), 118.8 (Ar), 121.9 (Ar), 124.5 (Ar), 129.1 (2 × Ar), 130.9 (Ar), 131.9 (2 × Ar), 136.9 (Ar), 148.1 (Ar), 156.9 (Ar). Anal. Calcd for C_17_H_19_BrO: C, 63.96; H, 6.00. Found: C, 63.99; H, 6.02.

4-isopropyl-1-methyl-2-((2-nitrobenzyl)oxy)benzene (**32**). White powder, 91% yield, mp 56–57 °C. ^1^H NMR (300 MHz, CDCl_3_): *δ* 1.25 (d, *J* = 7.2 Hz, 6H, 2 × CH_3_), 2.32 (s, 3H, ArCH_3_), 2.86–2.91 (m, 1H, CH), 5.50 (s, 2H, OCH_2_), 6.78 (s, 1H, Ar), 6.80–6.83 (dd, *J* = 1.2 Hz, *J* = 15 Hz, 1H, Ar), 7.13 (d, *J* = 7.8 Hz, 1H, Ar), 7.50–7.53 (m, 1H, Ar), 7.69–7.75 (m, 1H, Ar), 7.98–8.01 (m, 1H, Ar), 8.17–8.20 (m, 1H, Ar). ^13^C NMR (75 MHz, CDCl_3_): *δ* 16.1 (CH_3_), 24.1 (2 × CH_3_), 34.1 (CH), 66.6 (OCH_2_), 110.0 (Ar), 118.8 (Ar), 124.2 (Ar), 124.9 (Ar), 128.2 (Ar), 128.5 (Ar), 130.7 (Ar), 134.0 (Ar), 134.4 (Ar), 146.9 (Ar), 148.2 (Ar), 156.1 (Ar). Anal. Calcd for C_17_H_19_NO_3_: C, 71.56; H, 6.71; N, 4.91. Found: C, 71.71; H, 6.70; N, 4.92.

4-isopropyl-1-methyl-2-((3-nitrobenzyl)oxy)benzene (**33**). Yellow viscous oil, 72% yield. ^1^H NMR (400 MHz, CDCl_3_): *δ* 1.36 (d, *J* = 6.9 Hz, 6H, 2 × CH_3_), 2.38 (s, 3H, ArCH_3_), 2.96–3.00 (m, 1H, CH), 5.24 (s, 2H, OCH_2_), 6.88–6.91 (m, 2H, Ar), 7.19 (d, *J* = 7.5 Hz, 1H, Ar), 7.60–7.65 (m, 1H, Ar), 7.89 (d, *J* = 7.5 Hz, 1H, Ar), 8.23 (d, *J* = 7.5 Hz, 1H, Ar), 8.43 (s, 1H, Ar). ^13^C NMR (101 MHz, CDCl_3_): *δ* 16.1 (CH_3_), 24.2 (2 × CH_3_), 34.2 (CH), 68.5 (OCH_2_), 109.9 (Ar), 119.0 (Ar), 121.9 (Ar), 122.7 (Ar), 124.3 (Ar), 129.6 (Ar), 130.9, 133.1 (Ar), 139.9 (Ar), 148.1 (Ar), 148.4 (Ar), 156.3 (Ar). Anal. Calcd for C_17_H_19_NO_3_: C, 71.56; H, 6.71; N, 4.91. Found: C, 71.43; H, 6.73; N, 4.92.

4-isopropyl-1-methyl-2-((4-nitrobenzyl)oxy)benzene (**34**). Yellow amber powder, 95% yield, mp 88–95 °C. ^1^H NMR (300 MHz, CDCl_3_): *δ* 1.23 (d, *J* = 6.3 Hz, 6H, 2 × CH_3_), 2.28 (s, 3H, ArCH_3_), 2.84–2.88 (m, 1H, CH), 5.19 (s, 2H, CH_2_), 6.71 (s, 1H, Ar), 6.80 (d, *J* = 7.8 Hz, 1H, Ar), 7.11 (d, *J* =7.5 Hz, 1H, Ar), 7.64 (d, *J* = 8.1 Hz, 2H, Ar), 8.26 (d, *J* = 8.7 Hz, 2H, Ar). ^13^C NMR (75 MHz, CDCl_3_): *δ* 15.9 (CH_3_), 24.1 (2 × CH_3_), 34.1 (CH_3_), 68.5 (OCH_2_), 109.7 (Ar), 110.0 (Ar), 119.0 (2 × Ar), 123.8 (2 × Ar), 124.3 (Ar), 127.4 (Ar), 130.8 (Ar), 145.1 (Ar), 148.1 (C=O), 156.2 (C=O). Anal. Calcd for C_17_H_19_NO_3_: C, 71.56; H, 6.71; N, 4.91. Found: C, 71.63; H, 6.69; N, 4.90.

4-((5-isopropyl-2-methylphenoxy)methyl)aniline (**35**). Orange viscous oil, 70% yield. ^1^H NMR (300 MHz, CDCl_3_): *δ* 1.24–1.27 (m, 6H, 2 × CH_3_), 2.23 (s, 3H, ArCH_3_), 2.86–2.91 (m, 1H, CH), 3.86 (bs, 2H, NH_2_, D_2_O exch.), 4.97 (s, 2H, OCH_2_), 6.75–6.80 (m, 4H, Ar), 7.09 (d, *J* = 7.8 Hz, 1H, Ar), 7.28 (d, *J* = 8.7 Hz, 2H, Ar). ^13^C NMR (75 MHz, CDCl_3_): *δ* 16.1 (CH_3_), 24.2 (2 × CH_3_), 34.2 (CH), 70.1 (OCH_2_), 110.1 (Ar), 115.1 (2 × Ar), 118.2 (Ar), 124.5 (Ar), 124.6 (Ar), 128.9 (2 × Ar), 130.4 (2 × Ar), 130.8 (Ar), 146.0 (Ar), 147.8 (Ar), 157.1 (Ar). Anal. Calcd for C_17_H_21_NO: C, 79.96; H, 8.29; N, 5.49. Found: C, 80.14; H, 8.27; N, 5.51.

4-((5-isopropyl-2-methylphenoxy)methyl)benzonitrile (**36**). White powder, 79% yield, mp 74–76 °C. ^1^H NMR (300 MHz, CDCl_3_): *δ* 1.21–124 (m, 6H, 2 × CH_3_), 2.27 (s, 3H, ArCH_3_), 2.81–2.88 (m, 1H, CH), 5.14 (s, 2H, OCH_2_), 6.71 (s, 1H, Ar), 6.79 (d, *J* = 7.8 Hz, 1H, Ar), 7.11 (d, *J* = 7.5 Hz, 1H, Ar), 7.58 (d, *J* = 8.4 Hz, 2H, Ar), 7.70 (d, *J* = 8.1 Hz, 2H, Ar). ^13^C NMR (75 MHz, CDCl_3_): *δ* 16.0 (CH_3_), 24.1 (2 × CH_3_), 34.1 (CH), 68.9 (OCH_2_), 109.7 (Ar), 111.4 (CN), 118.8 (Ar), 124.3 (Ar), 127.3 (2 × Ar), 130.7 (Ar), 132.4 (2 × Ar), 143.1 (Ar), 148.1 (Ar), 156.2 (Ar). Anal. Calcd for C_18_H_19_NO: C, 81.47; H, 7.22; N, 5.28. Found: C, 71.56; H, 6.71; N, 4.91.

(4-((5-isopropyl-2-methylphenoxy)methyl)phenyl)(methyl)sulfane (**37**). White powder, 81% yield, mp 61–62 °C. ^1^H NMR (300 MHz, CDCl_3_): *δ* 1.35 (d, *J* = 6.9 Hz, 6H, 2 × CH_3_), 2.35 (s, 3H, ArCH_3_), 2.56 (s, 3H, SCH_3_), 2.92–2.99 (m, 1H, CH), 5.19 (s, 2H, OCH_2_), 6.86–6.88 (m, 2H, Ar), 7.18 (d, *J* = 8.1 Hz, 1H, Ar), 7.35–7.38 (m, 2H, Ar), 7.65 (d, *J* = 8.7 Hz, 2H, Ar). ^13^C NMR (75 MHz, CDCl_3_): *δ* 15.9 (SCH_3_), 16.2 (CH_3_), 24.4 (2 × CH_3_), 34.3 (CH), 69.6 (OCH_2_), 110.0 (Ar), 118.5 (Ar), 124.4 (Ar), 126.8 (2 × Ar), 128.0 (2 × Ar), 130.6 (Ar), 134.5 (Ar), 138.0 (Ar), 148.0 (Ar), 156.9 (Ar). Anal. Calcd for C_18_H_22_OS: C, 75.48; H, 7.74. Found: C, 75.37; H, 7.72.

4-isopropyl-1-methyl-2-((4-(methylsulfinyl)benzyl)oxy)benzene (**38**). Yellow viscous oil, 51% yield. ^1^H NMR (300 MHz, CDCl_3_): *δ* 1.24 (d, *J* = 6.3 Hz, 6H, 2 × CH_3_), 2.27 (s, 3H, ArCH_3_), 2.77 (s, 3H, SCH_3_), 2.85–2.87 (m, 1H, CH), 5.15 (s, 2H, OCH_2_), 6.75–6.80 (m, 2H, Ar), 7.09–7.12 (m, 1H, Ar), 7.65 (bs, 4H, Ar). ^13^C NMR (75 MHz, CDCl_3_): *δ* 16.0 (CH_3_), 24.1 (2 × CH_3_), 34.1 (CH), 44.7 (SCH_3_), 69.1 (OCH_2_), 109.9 (Ar), 118.7 (Ar), 124.1 (Ar), 124.3 (2 × Ar), 128.0 (2 × Ar), 130.7 (Ar), 141.2 (Ar), 145.0 (Ar), 148.0 (Ar), 156.4 (Ar). Anal. Calcd for C_18_H_22_O_2_S: C, 71.49; H, 7.33. Found: C, 71.35; H, 7.30.

4-isopropyl-1-methyl-2-((4-(methylsulfonyl)benzyl)oxy)benzene (**39**). Yellow viscous oil, 25% yield. ^1^H NMR (300 MHz, CDCl_3_) *δ* 1.21–1.22 (m, 6H, 2 × CH_3_), 2.27 (s, 3H, ArCH_3_); 2.84–2.86 (m, 1H, CH), 3.07 (s, 3H, SO_2_CH_3_); 5.17 (s, 2H, OCH_2_), 6.72 (s, 1H, Ar), 6.79 (d, *J* = 7.8 Hz, 1H, Ar), 7.09–7.12 (d, *J* = 7.5 Hz, 1H, Ar); 7.67 (d, *J* = 8.4 Hz, 2H, Ar), 7.97 (d, *J* = 8.1 Hz, 2H, Ar). Anal. Calcd for C_18_H_22_O_3_S: C, 67.89; H, 6.96. Found: C, 68.01; H, 6.99.

4-((5-isopropyl-2-methylphenoxy)methyl)-1,1′-biphenyl (**40**). White solid, 90% yield, mp = 99–100 °C. ^1^H NMR (400 MHz, CDCl_3_): *δ* 1.29–1.31 (d, *J* = 6.8 Hz, 6H, 2 × CH_3_), 2.33 (s, 3H, ArCH_3_), 2.89–2.96 (m, 1H, CH), 5.18 (s, 2H, OCH_2_), 6.81–6.85 (m, 2H, 2 × Ar), 7.14–7.16 (d, J = 7.6 Hz, 1H, Ar), 7.38–7.42 (m, 1H, Ar), 7.48–7.52 (m, 2H, 2 × Ar), 7.57–7.60 (d, *J* = 8.4 Hz, 2H, 2 × Ar), 7.65–7.68 (m, 4H, 4 × Ar). ^13^C NMR (101 MHz, CDCl_3_): *δ* 16.1 (CH_3_), 24.2 (2 × CH_3_), 34.2 (CH), 69.7 (OCH_2_), 110.1 (2 × Ar), 118.4 (2 × Ar), 124.5 (Ar), 127.2 (2 × Ar), 127.3 (2 × Ar), 127.4 (2 × Ar), 127.7 (2 × Ar), 128.8 (Ar), 130.6 (Ar), 126.7 (Ar), 148.0 (Ar), 156.9 (Ar). Anal. Calcd for C_23_H_24_O: C, 87.30; H, 7.64. Found: C, 87.47; H, 7.65.

1-((5-isopropyl-2-methylphenoxy)methyl)naphthalene (**41**). Yellow-brown sticky solid, 98% yield. ^1^H NMR (300 MHz, CDCl_3_): *δ* 1.29 (d, *J* = 7.2 Hz, 6H, 2 × CH_3_), 2.21 (s, 3H, ArCH_3_), 2.88–2.97 (m, 1H, CH), 5.52 (s, 2H, OCH_2_), 6.80–6.83 (m, 1H, Ar), 6.95–6.96 (m, 1H, Ar), 7.48 (s, 1H, Ar), 7.50 (m, 1H, Ar), 7.51–7.59 (m, 2H, Ar), 7.66 (d, *J* = 7.2 Hz, 1H, Ar) 7.86–7.94 (m, 2H, Ar), 8.10–8.14 (m, 1H, Ar). ^13^C NMR (75 MHz, CDCl_3_): *δ* 16.0 (CH_3_), 24.2 (2 × CH_3_), 34.2 (CH), 68.6 (OCH_2_), 110.0 (Ar), 118.4 (Ar), 123.9 (Ar), 124.6 (Ar), 125.3 (Ar), 125.8 (Ar), 126.2 (Ar), 126.3 (Ar), 128.6 (Ar), 128.8 (Ar), 130.6 (Ar), 131.6 (Ar), 132.9 (Ar), 133.7 (Ar), 148.0 (Ar), 156.9 (Ar). Anal. Calcd for C_21_H_22_O: C, 86.85; H, 7.64. Found: C, 86.97; H, 7.66.

2-((5-isopropyl-2-methylphenoxy)methyl)isoindoline-1,3-dione (**42**). Colourless viscous oil, 88% yield. ^1^H NMR (300 MHz, CDCl_3_): *δ* 1.23–1.26 (m, 6H, 2 × CH_3_), 2.18 (s, 3H, ArCH_3_), 2.80–2.93 (m, 1H, CH), 5.67 (s, 2H, OCH_2_), 6.79–6.82 (m, 1H, Ar), 7.06–7.04 (m, 1H, Ar), 7.74–7.78 (m, 2H, Ar), 7.88–7.92 (m, 2H, Ar). ^13^C NMR (75 MHz, CDCl_3_): *δ* 15.8 (CH_3_), 24.1 (2 × CH_3_), 34.0 (CH), 65.5 (OCH_2_), 112.6 (Ar), 120.3 (Ar), 123.8 (2 × Ar), 125.5 (Ar), 130.8 (Ar), 131.8 (Ar), 134.5 (2 × Ar), 148.0 (Ar), 154.3 (Ar), 167.2 (2 × C=O). Anal. Calcd for C_19_H_19_NO_3_: C, 73.77; H, 6.19; N, 4.53. Found: C, 73.69; H, 6.18; N, 4.55.

5-isopropyl-2-methylphenyl 6-methyl-2-oxo-2*H*-chromene-3-carboxylate (**43**). White powder, 76% yield, mp 122–124 °C. ^1^H NMR (300 MHz, CDCl_3_): *δ* 1.24 (d, *J* = 6.9 Hz, 6H, 2 × CH_3_), 2.22 (s, 3H, ArCH_3 carv._), 2.44 (s, 3H, ArCH_3 coum._), 2.89–2.95 (m, 1H, CH), 7.01–7.07 (m, 2H, Ar), 7.17–7.19 (m, 1H, Ar), 7.28–7.31 (m, 1H, Ar), 7.44–7.51 (m, 2H, Ar), 8.69 (s, 1H, =CH). ^13^C NMR (75 MHz, CDCl_3_): *δ* 16.0 (CH_3_), 20.8 (CH_3 coum._), 23.9 (2 × CH_3_), 33.6 (CH_3_), 116.7 (Ar), 117.4 (Ar), 117.6 (Ar), 119.8 (Ar), 124.5 (Ar), 127.2 (Ar), 129.4 (Ar), 131.0 (Ar), 134.8 (Ar), 136.0 (Ar), 148.2 (Ar), 149.0 (Ar), 149.9 (Ar), 153.6 (Ar), 156.8 (C=O), 161.6 (C=O). Anal. Calcd for C_21_H_20_O_4_: C, 74.98; H, 5.99. Found: C, 75.07; H, 6.00.

5-isopropyl-2-methylphenyl 6-chloro-2-oxo-2*H*-chromene-3-carboxylate (**44**). White powder, 70% yield, mp 114–115 °C. ^1^H NMR (300 MHz, CDCl_3_): *δ* 1.24 (d, *J* = 6.9 Hz, 6H, 2 × CH_3_), 2.21 (s, 3H, ArCH_3_), 2.87–2.92 (m, 1H, CH), 7.00 (s, 1H, Ar), 7.04–7.08 (m, 1H, Ar), 7.19 (d, *J* = 7.5 Hz, 1H, Ar), 7.34–7.37 (d, *J* = 8.1 Hz, 1H, Ar), 7.60–7.65 (m, 2H, Ar), 8.64 (s, 1H, =CH). ^13^C NMR (75 MHz, CDCl_3_): *δ* 15.9 (CH_3_), 23.9 (2 × CH_3_) 33.6 (CH), 118.4 (Ar), 118.7 (Ar), 118.8 (Ar), 119.6 (Ar), 124.7 (Ar), 127.1 (Ar), 128.7 (Ar), 130.3 (Ar), 131.0 (Ar), 134.6 (Ar), 148.2 (Ar), 148.3 (Ar), 148.9 (Ar), 153.7 (Ar), 155.8 (C=O), 161.1 (C=O). Anal. Calcd for C_20_H_17_ClO_4_: C, 67.33; H, 4.80. Found: C, 67.40; H, 4.79.

5-Isopropyl-2-methylphenyl 6-bromo-2-oxo-2*H*-chromene-3-carboxylate (**45**). White powder, 73% yield, mp 134–138 °C. ^1^H NMR (300 MHz, CDCl_3_): *δ* 1.24 (d, *J* = 6.3 Hz, 6H, 2 × CH_3_), 2.21 (s, 3H, ArCH_3_), 2.84–2.96 (m, 1H, CH), 7.00 (s, 1H, Ar), 7.05–7.08 (m, 1H, Ar), 7.19 (d, *J* = 7.5 Hz, 1H, Ar), 7.30 (d, *J* = 8.7 Hz, 1H, Ar), 7.74–7.80 (m, 2H, Ar), 8.64 (s, 1H, =CH coumarin). ^13^C NMR (75 MHz, CDCl_3_): *δ* 15.9 (CH_3_), 23.9 (2 × CH_3_), 33.6 (CH), 117.5 (Ar), 118.6 (Ar), 118.8 (Ar), 119.3 (Ar), 119.6 (Ar), 124.7 (Ar), 127.1 (Ar), 131.1 (Ar), 131.7 (Ar), 137.4 (Ar), 148.2 (Ar), 148.2 (Ar), 148.9 (Ar), 154.2 (Ar), 155.8 (C=O), 161.0 (C=O). Anal. Calcd for C_20_H_17_BrO_4_: C, 59.87; H, 4.27. Found: C, 59.92; H, 4.28.

2-(5-isopropyl-2-methylphenoxy)-*N*′-(3-nitrophenyl)acetohydrazide (**46**). White solid; 62% yield, mp = 127–128 °C. ^1^H NMR (400 MHz, CDCl_3_): *δ* 1.26–1.28 (d, *J* = 6.8 Hz, 6H, 2 × CH_3_), 2.34 (s, 3H, CH_3_), 2.88–2.95 (m, 1H, CH), 4.76 (s, 2H, OCH_2_), 6.41–6.42 (bs, 1H, NH, D_2_O exch.), 6.73 (s, 1H, Ar), 6.89–6.91 (d, *J* = 7.6 Hz, 1H, Ar), 7.15–7.18 (m, 2H, 2 × Ar), 7.37–7.39 (t, *J* = 8.0 Hz, 1H, Ar), 7.69–7.71 (m, 1H, Ar), 7.77–7.79 (m, 1H, Ar), 8.33–8.34 (bs, 1H, NH, D_2_O exch.). ^13^C NMR (101 MHz, CDCl_3_): *δ* 16.1 (CH_3_), 24.1 (2 × CH_3_), 34.1 (CH), 67.4 (OCH_2_), 108.0 (Ar), 109.9 (Ar), 116.0 (Ar), 119.1 (Ar), 120.2 (Ar), 123.8 (Ar), 130.0 (Ar), 131.2 (Ar), 148.7 (Ar), 148.9 (Ar), 149.2 (Ar), 155.1 (Ar), 169.0 (C=O). Anal. Calcd for C_18_H_21_N_3_O_4_: C, 62.96; H, 6.16; N, 12.24. Found: C, 63.05; H, 6.19; N, 12.29.

### 3.4. Crystal Structure Determination of Compound ***34***

C17H19NO3, M = 285.33, Monoclinic, space group P 21/c, a = 8.380(1), b = 15.416(1), c = 11.375(1)Å, β = 95.856(7), V = 1462.6(2)Å3, Z = 4 Dc = 1.296, µ = 0.089 mm^−1^, F(000) = 608. 9670 reflections were collected with a 4.356 < θ < 29.273 range with a completeness to theta 99.1%; 3395 were unique, the parameters were 190 and the final R index was 0.0619 for reflections having I > 2σI.

A light yellow prismatic shaped crystal (0.07 × 0.06 × 0.03) was used for data collection. Hydrogen atoms were all assigned in calculated positions and refined as isotropic. No relevant hydrogen bonds were detected. CCDC 1980137 contains the supplementary crystallographic data for this molecule. Data can be obtained free of charge from the Cambridge Crystallographic Data Centre [28]. Collection was carried out with a KM4 Xcalibur2 goniometer (Oxford Diffraction, Abingdon, UK) at 100 K. Mo/Kα radiation (40 mA/−40 KV), monochromated by an Oxford Diffraction Enhance ULTRA assembly, and an Oxford Diffraction Excalibur PX Ultra CCD were used for cells parameters determination and data collection. The integrated intensities, measured using the ω scan mode, were corrected for Lorentz and polarization effects [29]. Direct methods of SIR2004 [30] were used in solving the structure and the refinement was performed using the full-matrix least squares on F2 provided, within WinGX v.2013.3 routine [31], by SHELXL2014 [32]. Multi-scan symmetry-related measurement was used as experimental absorption correction type.

### 3.5. Anti-Helicobacter Pylori Activity

The MIC determination was performed by modified broth microdilution assay as previously described [33]. For MBC evaluation 10 µL of suspensions without visible growth were spotted on Skirrow agar plates surface and incubated for 72 h at 37 °C in microaerophilic conditions. The MBC was defined as the concentration that killed 99.9% of the initial inoculum.

### 3.6. Cell Lines and Treatments

The human adenocarcinoma gastric cell line (AGS), was derived from an untreated human adenocarcinoma of the stomach and retained the same cytological characteristics of the malignant cells obtained from Caucasian patients [34]. The AGS cells (ECACC 89090402) were obtained from CLS Cell Lines Services GmbH (Epplheim, Germany) and were cultured in Dulbecco’s Modified Eagle’s Medium (DMEM) supplemented with 4.5 g/L glucose, 2 mM L-glutamine and 10% Foetal Bovine Serum (FBS) (EuroClone S.p.A., Pero, Italy).

Working solutions of carvacrol and its derivatives (**6**, **9**, **16**, **17**, **20**, **21**, **29**, **32**–**35**, **38**, **39**, **42**–**45**) (600 mM) were freshly prepared in dimethyl sulfoxide (DMSO) and in DMEM according to the experimental design by serial dilutions in complete culture medium. The final concentration of DMSO in experiments was 0.14%. No toxicity on AGS cells was observed (data not shown). 5-Fluorouracil was used as positive control.

### 3.7. Cell Viability

Cell viability was tested by MTS (3-(4,5-dimethylthiazol-2-yl)-5-(3-carboxymethoxyphenyl)-2-(4-sulfophenyl)-2*H*-tetrazolium)) assay (Promega, Madison, WI, USA). The concentration of carvacrol and its derivatives for treatments was selected based on concentration—response curves constructed in preliminary experiments (data not shown). Briefly, AGS cells were seeded in 96-well plates (6 × 10^3^ cells/well) and treated for 24 h with different concentrations (50–800 μM) of specific molecules (5 replica wells for each treatment condition).

Briefly, cells were incubated with the MTS solution for at least 1 h and cell viability was determined colorimetrically by measuring the absorbance at 490 nm using GloMax-Multi Detection System (Promega, Madison, WI, USA). Cell viability was expressed as the percentage compared with the untreated cells designated as 100%. The IC_50_ value was calculated from the concentration-response curves by nonlinear regression analysis [35].

### 3.8. Statistical Analysis

A *p* value of 0.05 was considered statistically significant. IC_50_ values was calculated using the GraphPad Prism 7 software.

## 4. Conclusions

Based on the shortlisted hits, a large series of carvacrol-based molecules were designed, synthesized and evaluated for their ability to act as dual agents (inhibitory action against the growth of *H. pylori* and AGS cells) along with the assessment of robust structure-activity relationships. Several hits with required balance of activities were extrapolated as a result of this study. Moreover, the most active compounds displayed antimicrobial activity with similar MIC and MBC values toward *H. pylori* strains with a different antibiotic susceptibility pattern, thus suggesting a mechanism of action alternative to metronidazole, amoxicillin and clarithromycin. The most important result is that the anti-*Helicobacter pylori* activity was not only strictly related to the presence of an OH moiety, as reported for the general antibacterial activity of the parent compound. Among the compounds evaluated, some analogues exhibited MIC/MBC values in the low µg/mL range. In this regard, the most noteworthy compounds displaying the lowest MIC/MBC activity against *H. pylori,* such as compounds **16** and **39**, also showed good activity against AGS cells (IC_50_ compound **16** = 209 μM; IC_50_ compound **39** = 209 μM). Further studies might explain the potential synergistic effects of the combination of these derivatives with the currently used therapeutics. In addition, the possibility to treat drug resistant *H. pylori* strains would be beneficial in the clinical setting due to the high development of resistance attributed to *H. pylori*. Finally, whereas the development of biofilms by *H. pylori* as well as the ability of the microorganism to enter the Viable But Non-Culturable (VBNC) state represent two different survival strategies that induce resistance/tolerance of the microorganism or the microbial community to antimicrobial drugs [36], future studies will be carried out to evaluate the potential activity of such molecules on both *H. pylori* biofilm and VBNC state.

## Figures and Tables

**Table 1:** MIC and MBC values for carvacrol (parent compound) and its semi-synthetic derivatives (**1**–**46**) against nine strains of *H. pylori*. Antibiotic susceptibility is reported for each *H. pylori* strain.

*H. pylori* Strains MIC/MBC (µg/mL)									
	F4	190	23	110 R	NCTC 11637	F1	F34/497	F40/499	F40/442
Carvacrol	64/64	64/64	64/64	64/64	64/64	64/64	16/32	32/64	32/64
**1**	128/128	>128/>128	>128/>128	128/128	>128/>128	128/128	128/128	64/64	64/64
**2**	64/64	64/128	>128/>128	64/64	128/>128	64/64	32/64	32/64	64/64
**3**	64/128	64/64	128/>128	64/64	64/64	64/64	32/64	64/64	64/64
**4**	128/128	128/128	128/128	128/128	128/128	128/128	128/128	128/128	128/128
**5**	64/64	64/64	64/64	64/64	128/128	64/64	64/64	64/64	64/64
**6**	16/32	32/32	32/64	64/128	64/64	32/32	32/32	16/16	32/32
**7**	32/32	32/64	64/128	64/128	64/64	32/32	32/32	64/64	64/64
**8**	64/64	64/64	64/64	64/128	64/64	64/64	64/64	64/64	64/64
**9**	8/8	32/32	32/64	32/32	32/32	16/16	8/8	16/16	16/16
**10**	128/128	128/128	128/128	128/128	128/128	128/128	128/128	128/128	128/128
**11**	128/128	128/128	128/128	128/128	128/128	128/128	128/128	128/128	128/128
**12**	64/64	32/32	128/128	64/64	64/64	64/64	64/64	32/64	64/64
**13**	>128/>128	>128/>128	>128/>128	>128/>128	>128/>128	>128/>128	128/128	128/128	128/128
**14**	64/64	64/64	64/64	64/64	64/64	64/64	64/64	64/64	64/64
**15**	64/128	64/64	32/32	64/64	64/64	32/64	32/32	32/32	32/32
**16**	8/16	8/16	32/32	8/8	32/32	4/8	2/2	8/8	4/8
**17**	32/32	32/32	16/16	32/32	32/32	16/16	8/32	16/16	16/16
**18**	32/32	32/64	32/32	64/128	32/64	16/32	16/16	16/16	16/16
**19**	32/64	>128/>128	>128/>128	>128/>128	128/128	32/32	16/32	64/128	64/128
**20**	16/32	128/128	32/64	128/>128	64/64	32/32	16/32	64/64	64/64
**21**	16/32	32/32	32/64	32/64	32/64	32/32	16/32	32/32	32/32
**22**	>128/>128	>128/>128	>128/>128	>128/>128	>128/>128	128/>128	>128/>128	>128/>128	>128/>128
**23**	64/64	32/32	64/64	32/64	64/64	64/64	64/64	32/32	32/64
**24**	32/32	64/128	32/32	64/64	64/128	32/32	128/128	32/32	32/32
**25**	64/64	32/32	64/64	64/64	64/64	64/64	32/64	32/64	32/64
**26**	32/32	32/32	32/32	32/32	32/64	16/16	32/64	16/16	16/16
**27**	32/64	32/32	64/64	32/32	64/64	32/64	32/32	32/32	32/32
**28**	16/16	16/16	32/64	16/32	16/32	8/8	32/64	8/8	8/16
**29**	16/64	32/64	64/64	64/64	64/64	64/64	16/32	64/64	16/32
**30**	32/64	64/128	64/128	64/128	64/128	64/64	32/32	32/64	32/64
**31**	32/32	16/32	32/64	16/16	64/64	16/32	64/64	8/16	16/32
**32**	16/32	64/64	64/64	64/64	32/64	32/32	16/16	32/64	16/32
**33**	8/8	4/4	8/8	4/4	4/4	8/8	8/8	8/8	8/8
**34**	8/8	4/4	4/4	4/8	4/4	4/4	4/4	4/4	4/4
**35**	4/8	16/16	32/64	16/16	32/64	4/4	2/2	16/16	16/16
**36**	8/16	4/4	8/8	8/8	8/8	8/8	8/8	8/8	8/8
**37**	32/64	64/64	64/64	64/64	64/64	32/64	32/32	64/64	32/64
**38**	8/8	4/4	8/8	4/4	8/8	4/4	2/2	8/8	8/8
**39**	2/2	4/4	2/2	4/4	2/2	2/2	2/2	2/2	2/2
**40**	8/8	8/16	8/16	8/16	8/16	4/4	4/4	4/4	4/4
**41**	>128/>128	>128/>128	>128/>128	>128/>128	>128/>128	>128/>128	>128/>128	>128/>128	>128/>128
**42**	16/16	16/32	32/32	32/32	32/32	32/32	16/32	32/32	16/16
**43**	32/32	64/128	128/>128	128/>128	128/>128	32/32	64/128	32/32	32/32
**44**	32/32	32/32	32/32	32/32	32/32	32/32	32/32	32/32	32/32
**45**	32/32	32/128	64/>128	32/128	64/128	32/32	32/32	32/32	32/32
**46**	64/128	32/64	32/64	32/64	32/64	64/128	64/>128	32/64	64/128
Antibiotic susceptibility	MTZ+	MTZ−	MTZ−	MTZ+	MTZ+	MTZ−	MTZ+	MTZ+	MTZ+
	CLR+	CLR−	CLR−	CLR−	CLR−	CLR+	CLR+	CLR+	CLR−
	AMX−	AMX−	AMX−	AMX−	AMX−	AMX−	AMX−	AMX−	AMX−

MTZ+ = metronidazole resistant (MIC > 8 µg/mL); MTZ− = metronidazole susceptible (MIC ≤ 8 µg/mL); CLR+ = clarithromycin resistant (MIC > 0.25 µg/mL); CLR− = clarithromycin susceptible (MIC ≤ 0.25 µg/mL); AMX+ = amoxicillin resistant (MIC > 0.125 µg/mL); AMX− = amoxicillin susceptible (MIC ≤ 0.125 µg/mL). Compounds selected for in vitro anti-proliferative activity assays against AGS cells are highlighted in grey.

**Table 2:** IC_50_ values are expressed as mean ± standard deviation (SD) of three experiments with quintuplicate determinations.

Compound	IC_50_ (μM) ^a^
Carvacrol	300 ± 6.5
**6**	530 ± 16
**9**	527 ± 8.0
**16**	209 ± 9.4
**17**	366 ± 13
**20**	na
**21**	179 ± 9.0
**29**	na
**32**	382 ± 13
**33**	615 ± 9.6
**34**	na
**35**	283 ± 12
**38**	217 ± 7.6
**39**	209 ± 10
**42**	722 ± 8.5
**43**	na
**44**	na
**45**	na
5-Fluorouracil (5-FU)	82.3 ± 5.6

^a^ Data are expressed as mean ± SD, *n* = 3; na: not active at the maximum concentration tested (800 μM).

## Supplementary Materials

The following are available online at https://www.mdpi.com/1424-8247/13/11/405/s1. Table S1: Crystal data and structure refinement for compound **34**, Table S2: Atomic coordinates (x 104) and equivalent isotropic displacement parameters (Å2x 103) for compound **34**. U(eq) is defined as one third of the trace of the orthogonalized Uij tensor, Table S3: Bond lengths [Å] and angles [°] for compound **34**, Table S4**:** Anisotropic displacement parameters (Å2x 103) for compound **34**. The anisotropic displacement factor exponent takes the form: −2 2[h2a*2U11 + … + 2 h k a* b* U12]. ^1^H, ^13^C and ^19^F NMR spectra of new compounds.
