# Supplementary material for: Synthesis and Biological Evaluation of Carvacrol-Based Derivatives as Dual Inhibitors of H. pylori Strains and AGS Cell Proliferation
Source: Pharmaceuticals (Basel). 2020 Nov 19;13(11):405. doi: 10.3390/ph13110405 (PMC7699384; doi:10.3390/ph13110405)
Supplement: Supplementary file 1 [file pharmaceuticals-13-00405-s001.pdf]

Synthesis and biological evaluation of carvacrol-based derivatives as dual inhibitors of *H. pylori* strains and AGS cell proliferation

Francesca Sisto[1], Simone Carradori[2,*], Paolo Guglielmi[3], Carmen Beatrice Traversi[2], Mattia Spano[3], Anatoly P. Sobolev[4], Daniela Secci[3], Maria Carmela Di Marcantonio[5], Entela Haloci[6], Rossella Grande[2], Gabriella Mincione[5]

[1] Department of Biomedical, Surgical and Dental Sciences, University of Milan, 20122 Milan, Italy; francesca.sisto@unimi.it (F.S.)
[2] Department of Pharmacy, "G. d'Annunzio" University of Chieti-Pescara, Via dei Vestini 31, 66100 Chieti, Italy; simone.carradori@unich.it (S.C.); cbtraversi@gmail.com (C.B.T.); rossella.grande@unich.it (R.G.)
[3] Department of Chemistry and Technology of Drugs, Sapienza University of Rome, P.le A. Moro 5, 00185 Rome, Italy; paolo.guglielmi@uniroma1.it (P.G.); mattia.spano@uniroma1.it (M.S.); daniela.secci@uniroma1.it (D.S.)
[4] Institute for Biological Systems, "Annalaura Segre" Magnetic Resonance Laboratory, CNR, 00015 Monterotondo (Rome), Italy; anatoly.sobolev@cnr.it (A.P.S.)
[5] Department of Medical, Oral, and Biotechnological Sciences, "G. d'Annunzio" University of Chieti-Pescara, Chieti, Italy; dimarcantonio@unich.it (M.C.D.M.); gabriella.mincione@unich.it (G.M.)
[6] Department of Pharmacy, University of Medicine, Tirana, Rr. Dibres 369, Tirana, Albania; entela.haloci@umed.edu.al (E.H.)
* Correspondence: simone.carradori@unich.it (S.C.)

Table S1. Crystal data and structure refinement for compound **34**.

Identification code	exp_139	
Empirical formula	C17 H19 N O3	
Formula weight	285.33	
Temperature	100(2) K	
Wavelength	0.71073 Å	
Crystal system	Monoclinic	
Space group	P 21/c	
Unit cell dimensions	a = 8.380(1) Å	α= 90°.
	b = 15.416(1) Å	β= 95.542(4)°.
	c = 11.375(1) Å	γ = 90°.
Volume	1462.6(2) $Å^3$	
Z	4	
Density (calculated)	1.296 Mg/m^3	
Absorption coefficient	0.089 mm^{-1}	
F(000)	608	
Crystal size	0.07 x 0.06 x 0.03 mm^3	
Theta range for data collection	4.356 to 29.273°.	
Index ranges	-11<=h<=10, -18<=k<=20, -15<=l<=15	
Reflections collected	9670	
Independent reflections	3395 [R(int) = 0.0335]	
Completeness to theta = 25.242°	99.1 %	
Refinement method	Full-matrix least-squares on F^2	
Data / restraints / parameters	3395 / 0 / 190	
Goodness-of-fit on F^2	1.040	
Final R indices [I>2sigma(I)]	R1 = 0.0619, wR2 = 0.1549	
R indices (all data)	R1 = 0.0884, wR2 = 0.1757	
Extinction coefficient	n/a	
Largest diff. peak and hole	0.606 and -0.632 e.$Å^{-3}$	

Table S2. Atomic coordinates (x 10^4) and equivalent isotropic displacement parameters (Å^{2}x 10^3) for compound **34**. U(eq) is defined as one third of the trace of the orthogonalized U^{ij} tensor.

	x	y	z	U(eq)
N(1)	4419(2)	1925(1)	4344(2)	23(1)
O(1)	7212(2)	230(1)	-142(1)	21(1)
O(2)	3973(2)	2663(1)	4079(1)	31(1)
O(3)	4265(2)	1595(1)	5312(1)	36(1)
C(1)	5180(2)	1408(1)	3473(2)	19(1)
C(2)	5996(3)	664(1)	3850(2)	21(1)
C(3)	6712(3)	175(1)	3030(2)	21(1)
C(4)	6592(2)	421(1)	1845(2)	18(1)
C(5)	5745(2)	1168(1)	1491(2)	19(1)
C(6)	5045(2)	1675(1)	2309(2)	19(1)
C(7)	7389(3)	-146(1)	1001(2)	19(1)
C(8)	7915(2)	-198(1)	-1024(2)	18(1)
C(9)	7745(2)	214(1)	-2129(2)	19(1)
C(10)	8433(3)	-196(1)	-3038(2)	22(1)
C(11)	9253(3)	-977(1)	-2883(2)	22(1)
C(12)	9413(2)	-1381(1)	-1786(2)	20(1)
C(13)	8728(2)	-981(1)	-856(2)	19(1)
C(14)	6866(3)	1063(1)	-2291(2)	23(1)
C(15)	10282(3)	-2245(1)	-1636(2)	21(1)
C(16)	9165(3)	-2991(2)	-2051(2)	33(1)
C(17)	11042(3)	-2413(2)	-386(2)	30(1)

Table S3. Bond lengths [Å] and angles [°] for compound **34**.

N(1)-O(2)	1.226(2)
N(1)-O(3)	1.230(2)
N(1)-C(1)	1.465(3)
O(1)-C(8)	1.380(2)
O(1)-C(7)	1.419(2)
C(1)-C(6)	1.381(3)
C(1)-C(2)	1.383(3)
C(2)-C(3)	1.381(3)
C(2)-H(2)	0.9300
C(3)-C(4)	1.395(3)
C(3)-H(3)	0.9300
C(4)-C(5)	1.391(3)
C(4)-C(7)	1.502(3)
C(5)-C(6)	1.388(3)
C(5)-H(5)	0.9300
C(6)-H(6)	0.9300
C(7)-H(7A)	0.9700
C(7)-H(7B)	0.9700
C(8)-C(13)	1.390(3)
C(8)-C(9)	1.403(3)
C(9)-C(10)	1.384(3)
C(9)-C(14)	1.505(3)
C(10)-C(11)	1.390(3)
C(10)-H(10)	0.9300
C(11)-C(12)	1.388(3)
C(11)-H(11)	0.9300
C(12)-C(13)	1.395(3)
C(12)-C(15)	1.521(3)
C(13)-H(13)	0.9300
C(14)-H(14A)	0.9600
C(14)-H(14B)	0.9600
C(14)-H(14C)	0.9600
C(15)-C(17)	1.523(3)
C(15)-C(16)	1.528(3)

C(15)-H(15)	0.9800
C(16)-H(16A)	0.9600
C(16)-H(16B)	0.9600
C(16)-H(16C)	0.9600
C(17)-H(17A)	0.9600
C(17)-H(17B)	0.9600
C(17)-H(17C)	0.9600
O(2)-N(1)-O(3)	123.24(18)
O(2)-N(1)-C(1)	118.68(18)
O(3)-N(1)-C(1)	118.08(19)
C(8)-O(1)-C(7)	117.15(16)
C(6)-C(1)-C(2)	122.50(19)
C(6)-C(1)-N(1)	119.08(19)
C(2)-C(1)-N(1)	118.42(18)
C(3)-C(2)-C(1)	118.55(19)
C(3)-C(2)-H(2)	120.7
C(1)-C(2)-H(2)	120.7
C(2)-C(3)-C(4)	120.6(2)
C(2)-C(3)-H(3)	119.7
C(4)-C(3)-H(3)	119.7
C(5)-C(4)-C(3)	119.47(19)
C(5)-C(4)-C(7)	122.89(18)
C(3)-C(4)-C(7)	117.64(19)
C(6)-C(5)-C(4)	120.60(19)
C(6)-C(5)-H(5)	119.7
C(4)-C(5)-H(5)	119.7
C(1)-C(6)-C(5)	118.3(2)
C(1)-C(6)-H(6)	120.9
C(5)-C(6)-H(6)	120.9
O(1)-C(7)-C(4)	109.51(17)
O(1)-C(7)-H(7A)	109.8
C(4)-C(7)-H(7A)	109.8
O(1)-C(7)-H(7B)	109.8
C(4)-C(7)-H(7B)	109.8
H(7A)-C(7)-H(7B)	108.2

O(1)-C(8)-C(13)	123.62(18)
O(1)-C(8)-C(9)	114.89(19)
C(13)-C(8)-C(9)	121.49(19)
C(10)-C(9)-C(8)	116.7(2)
C(10)-C(9)-C(14)	122.66(19)
C(8)-C(9)-C(14)	120.62(18)
C(9)-C(10)-C(11)	122.44(19)
C(9)-C(10)-H(10)	118.8
C(11)-C(10)-H(10)	118.8
C(12)-C(11)-C(10)	120.44(19)
C(12)-C(11)-H(11)	119.8
C(10)-C(11)-H(11)	119.8
C(11)-C(12)-C(13)	118.2(2)
C(11)-C(12)-C(15)	119.83(18)
C(13)-C(12)-C(15)	121.95(19)
C(8)-C(13)-C(12)	120.72(19)
C(8)-C(13)-H(13)	119.6
C(12)-C(13)-H(13)	119.6
C(9)-C(14)-H(14A)	109.5
C(9)-C(14)-H(14B)	109.5
H(14A)-C(14)-H(14B)	109.5
C(9)-C(14)-H(14C)	109.5
H(14A)-C(14)-H(14C)	109.5
H(14B)-C(14)-H(14C)	109.5
C(12)-C(15)-C(17)	113.97(18)
C(12)-C(15)-C(16)	110.65(18)
C(17)-C(15)-C(16)	110.4(2)
C(12)-C(15)-H(15)	107.1
C(17)-C(15)-H(15)	107.1
C(16)-C(15)-H(15)	107.1
C(15)-C(16)-H(16A)	109.5
C(15)-C(16)-H(16B)	109.5
H(16A)-C(16)-H(16B)	109.5
C(15)-C(16)-H(16C)	109.5
H(16A)-C(16)-H(16C)	109.5
H(16B)-C(16)-H(16C)	109.5

C(15)-C(17)-H(17A)	109.5
C(15)-C(17)-H(17B)	109.5
H(17A)-C(17)-H(17B)	109.5
C(15)-C(17)-H(17C)	109.5
H(17A)-C(17)-H(17C)	109.5
H(17B)-C(17)-H(17C)	109.5

Table S4. Anisotropic displacement parameters ($Å^2 x 10^3$) for compound **34**. The anisotropic displacement factor exponent takes the form: $-2\pi^2[h^2a^{*2}U^{11} + ... + 2 h k a^* b^* U^{12}]$.

	U^{11}	U^{22}	U^{33}	U^{23}	U^{13}	U^{12}
N(1)	23(1)	25(1)	20(1)	-5(1)	1(1)	0(1)
O(1)	27(1)	19(1)	18(1)	0(1)	7(1)	3(1)
O(2)	41(1)	22(1)	30(1)	-4(1)	4(1)	7(1)
O(3)	50(1)	39(1)	20(1)	1(1)	12(1)	13(1)
C(1)	19(1)	18(1)	20(1)	-4(1)	3(1)	-1(1)
C(2)	24(1)	23(1)	17(1)	2(1)	1(1)	0(1)
C(3)	23(1)	18(1)	24(1)	1(1)	3(1)	2(1)
C(4)	18(1)	17(1)	19(1)	-2(1)	4(1)	-3(1)
C(5)	21(1)	20(1)	18(1)	1(1)	4(1)	-2(1)
C(6)	21(1)	16(1)	21(1)	1(1)	2(1)	1(1)
C(7)	23(1)	18(1)	19(1)	1(1)	6(1)	1(1)
C(8)	17(1)	19(1)	18(1)	-3(1)	4(1)	-4(1)
C(9)	19(1)	17(1)	21(1)	0(1)	3(1)	-5(1)
C(10)	24(1)	23(1)	17(1)	1(1)	2(1)	-3(1)
C(11)	23(1)	23(1)	20(1)	-6(1)	7(1)	-4(1)
C(12)	18(1)	18(1)	23(1)	-4(1)	3(1)	-3(1)
C(13)	21(1)	18(1)	19(1)	0(1)	4(1)	-2(1)
C(14)	25(1)	20(1)	22(1)	1(1)	3(1)	1(1)
C(15)	21(1)	20(1)	24(1)	-4(1)	7(1)	0(1)
C(16)	28(1)	22(1)	50(2)	-7(1)	4(1)	2(1)
C(17)	36(1)	25(1)	29(1)	2(1)	5(1)	7(1)

^{1}H spectrum of compound 1

^{13}C spectrum of compound 1

^{1}H spectrum of compound 2

^{13}C spectrum of compound 2

^{1}H spectrum of compound 3

^{13}C spectrum of compound 3

^{1}H spectrum of compound 4

^{13}C spectrum of compound 4

^{1}H spectrum of compound 5

^{13}C spectrum of compound 5

^{1}H spectrum of compound 6

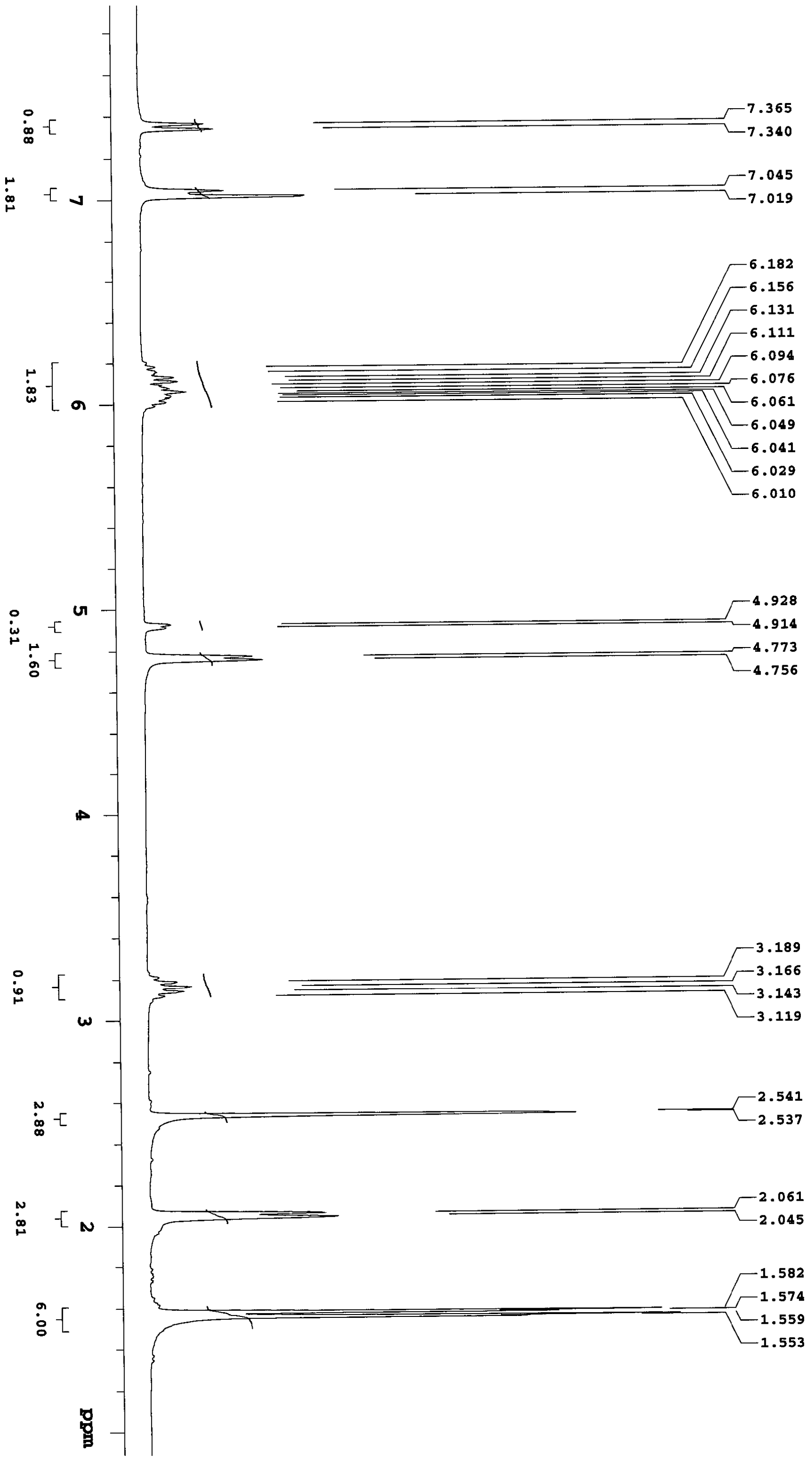

^{13}C spectrum of compound 6

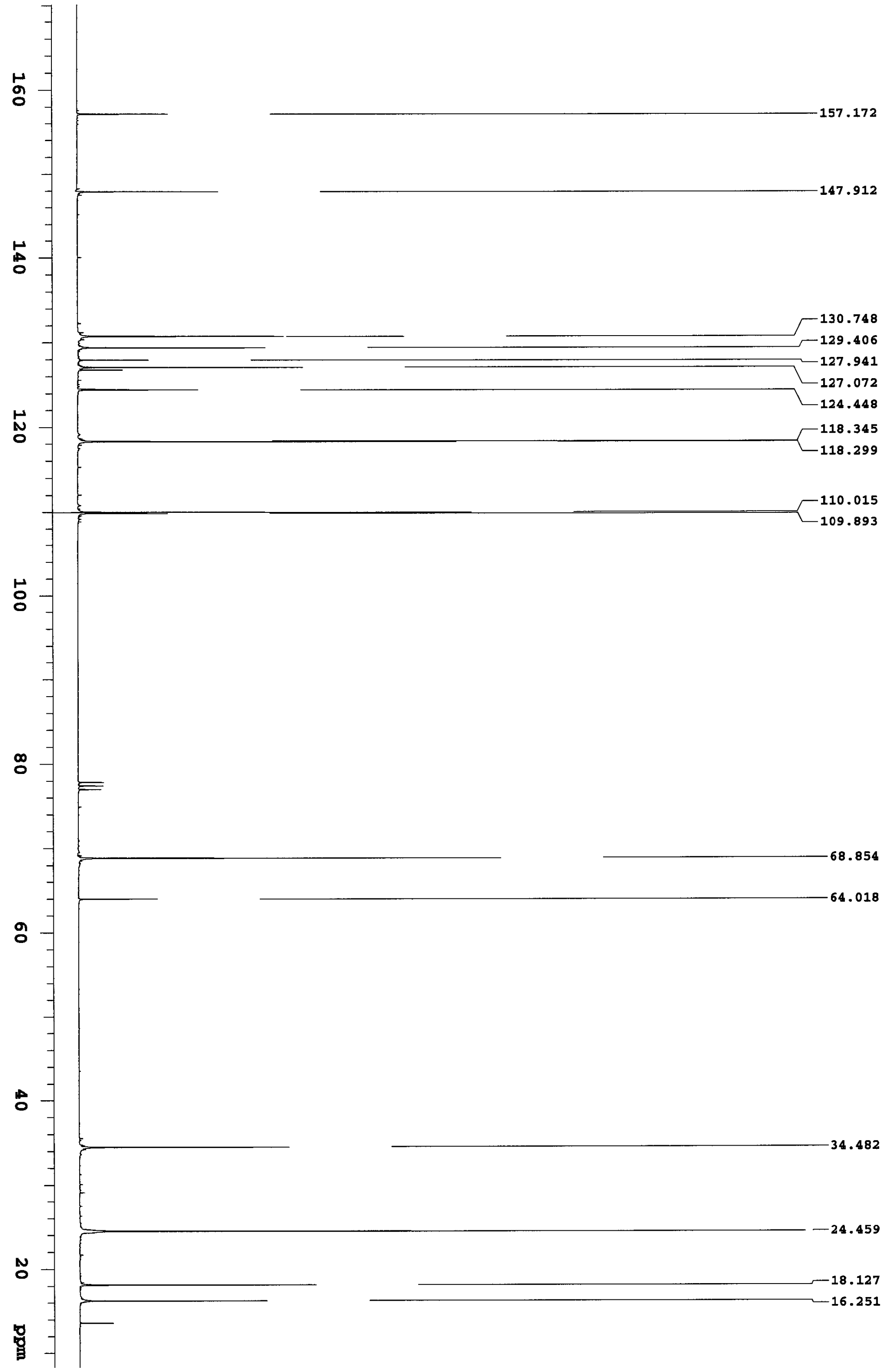

^{1}H spectrum of compound 7

^{13}C spectrum of compound 7

^{1}H spectrum of compound 8

^{13}C spectrum of compound 8

^{1}H spectrum of compound 10

^{13}C spectrum of compound 10

^{1}H spectrum of compound 11

^{13}C spectrum of compound 11

^{1}H spectrum of compound 14

^{13}C spectrum of compound 14

^{1}H spectrum of compound 16

^{13}C spectrum of compound 16

^{1}H spectrum of compound 17

^{13}C spectrum of compound 17

^{1}H-NMR of compound 18

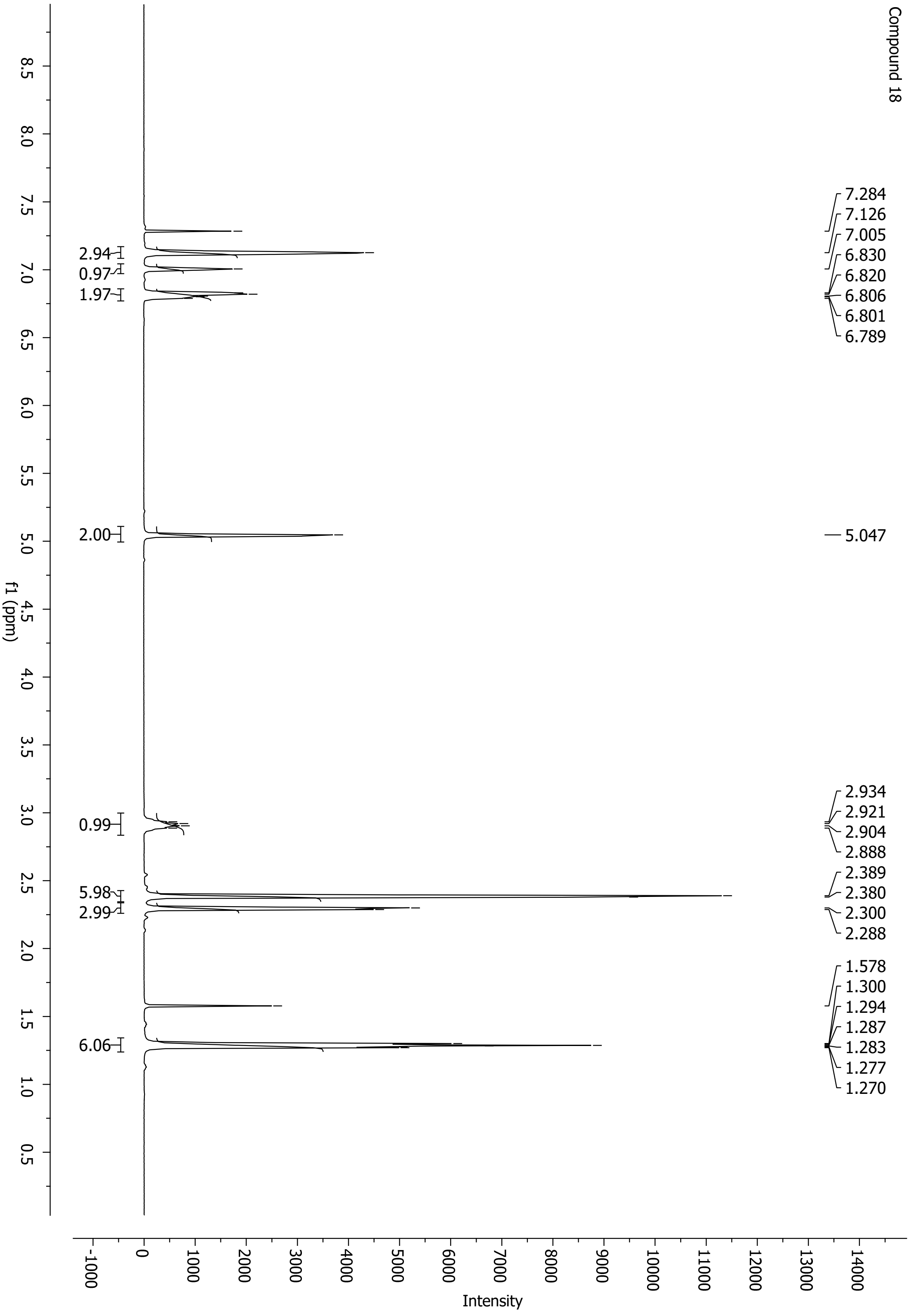

^{13}C-NMR of compound 18

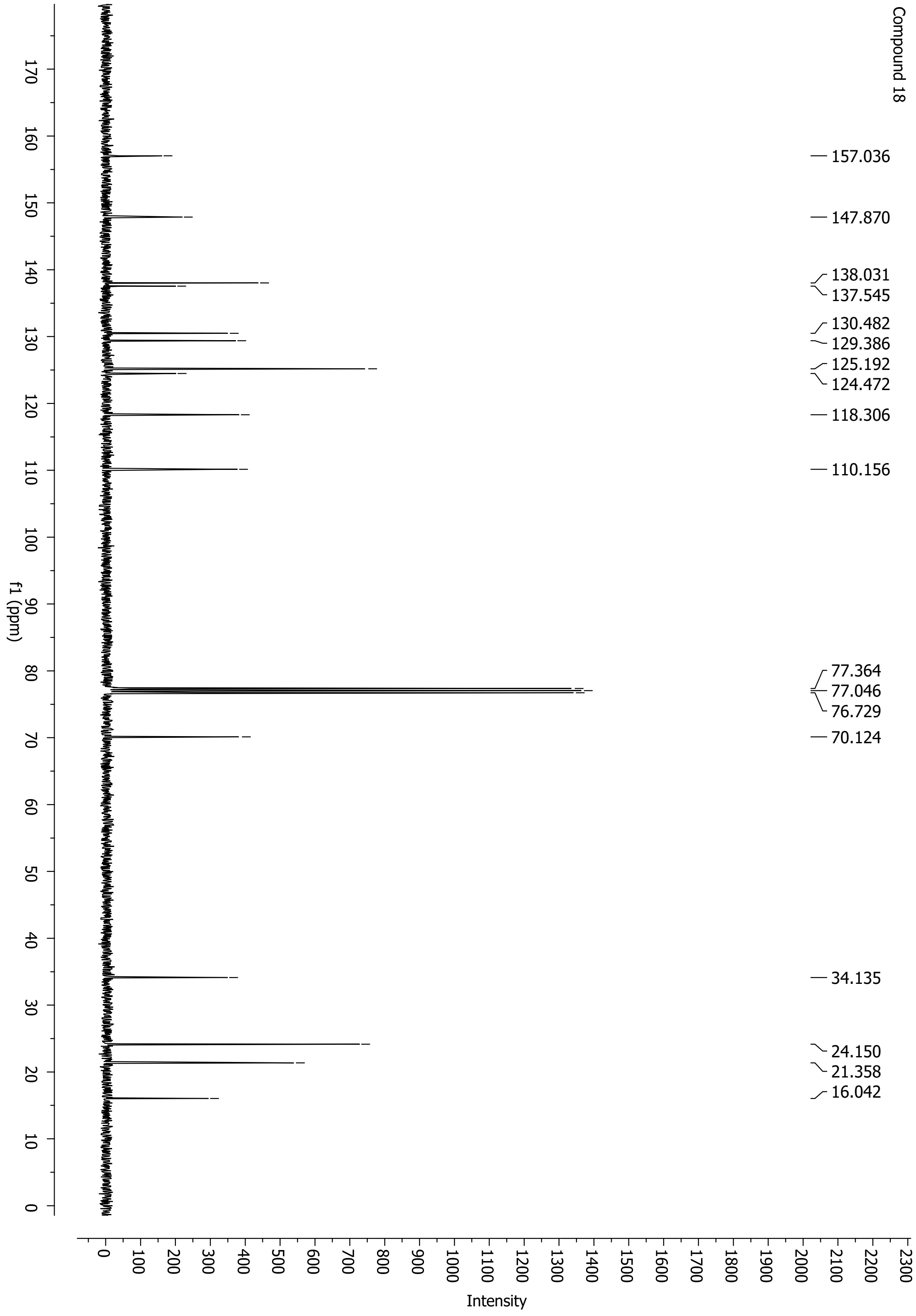

^{1}H spectrum of compound 19

^{13}C spectrum of compound 19

^{19}F spectrum of compound 19

^{1}H spectrum of compound 20

^{13}C spectrum of compound 20

^{19}F spectrum of compound 20

^{1}H spectrum of compound 21

^{13}C spectrum of compound 21

^{19}F spectrum of compound 21

^{1}H-NMR of compound 22

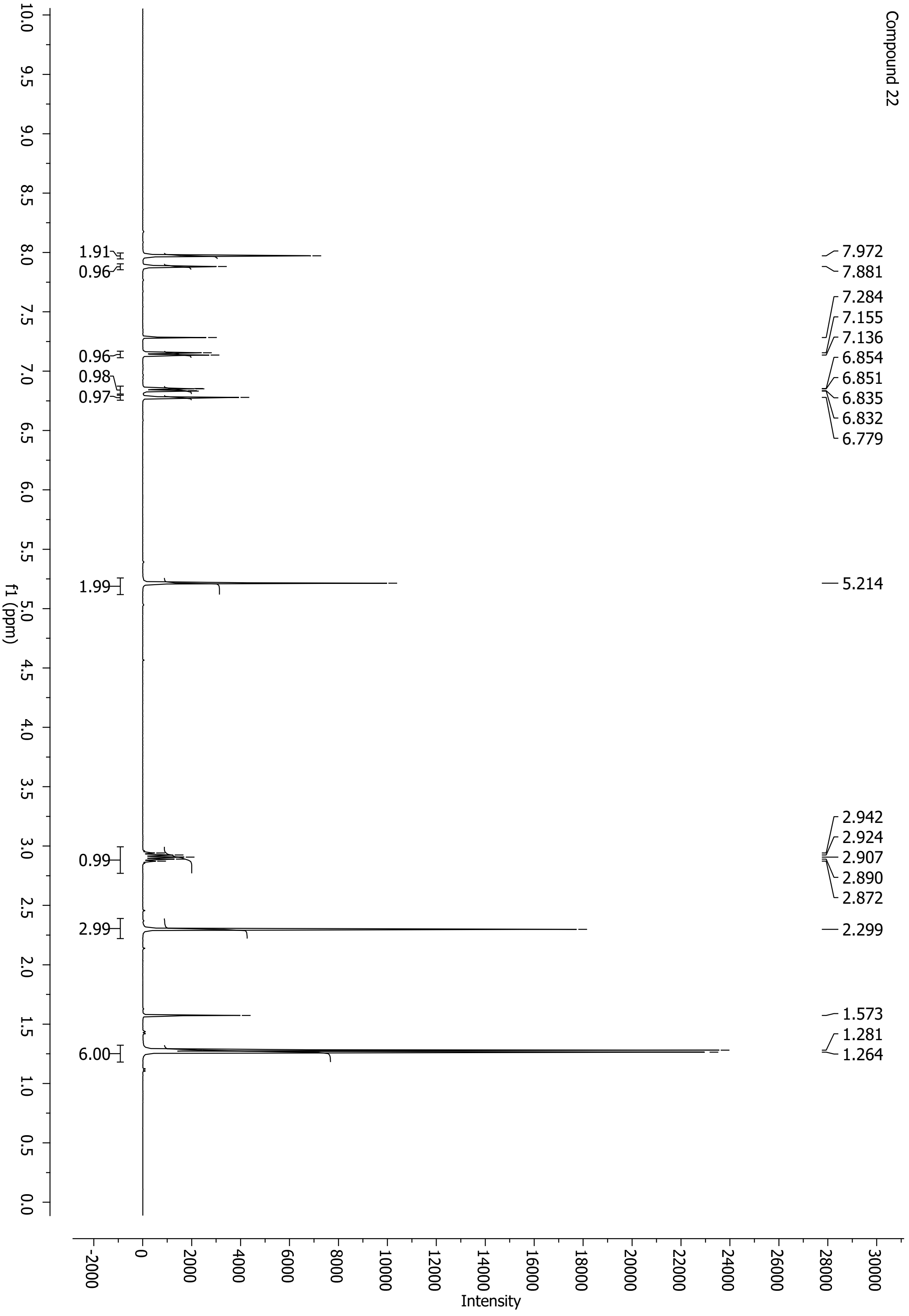

^{13}C-NMR of compound 22

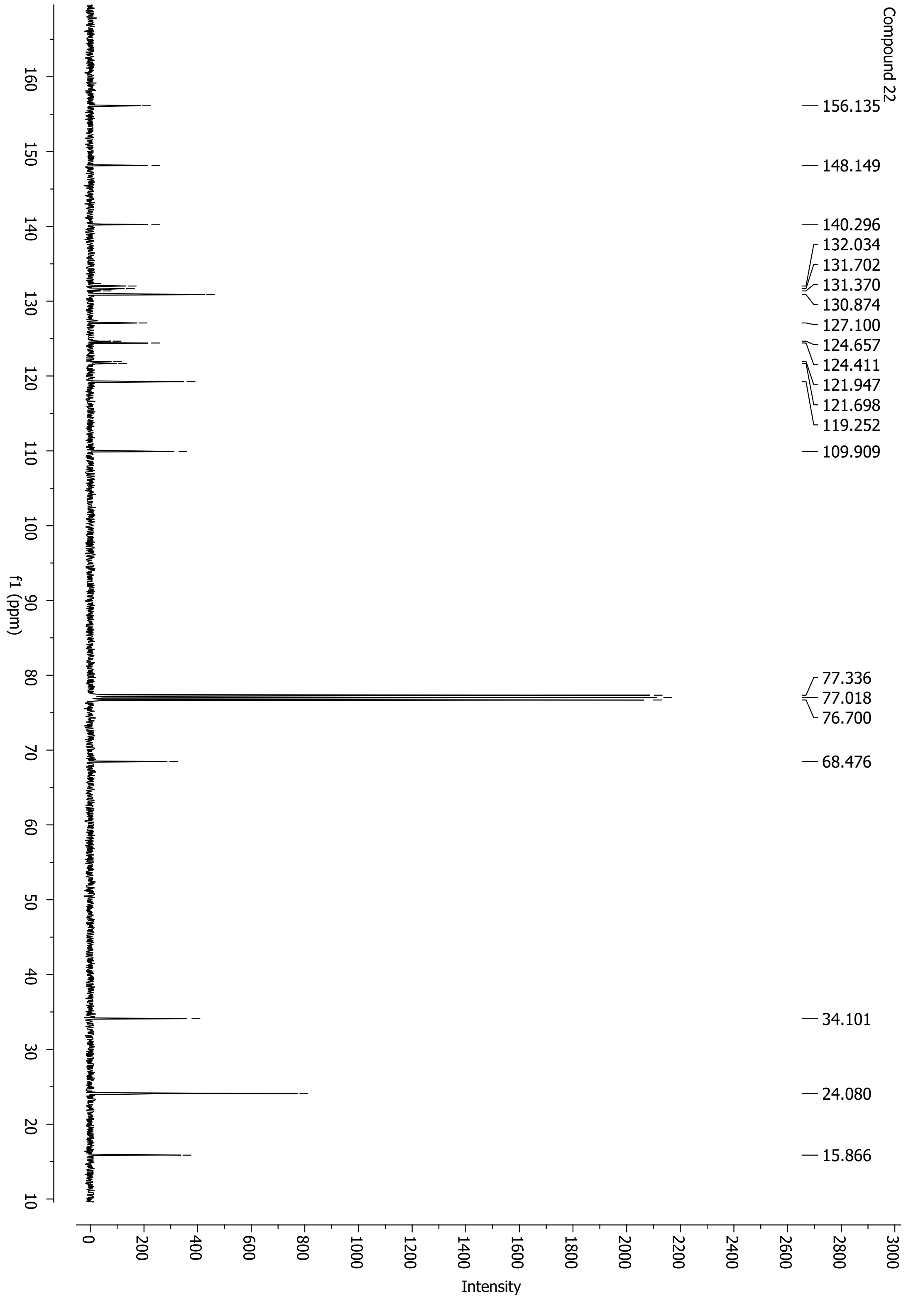

^{1}H spectrum of compound 23

^{13}C spectrum of compound 23

^{19}F spectrum of compound 23

^{1}H-NMR of compound 24

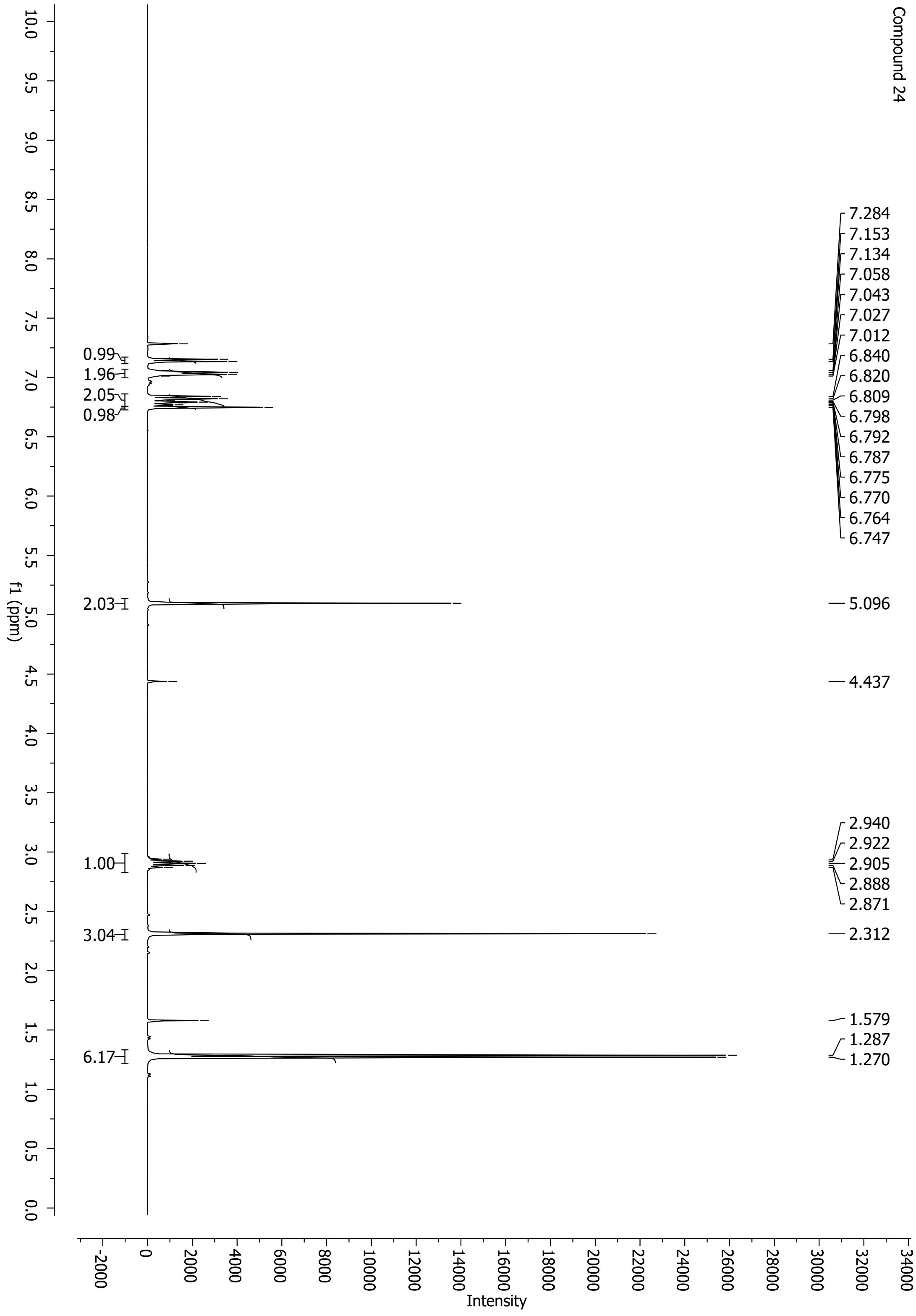

^{13}C-NMR of compound 24

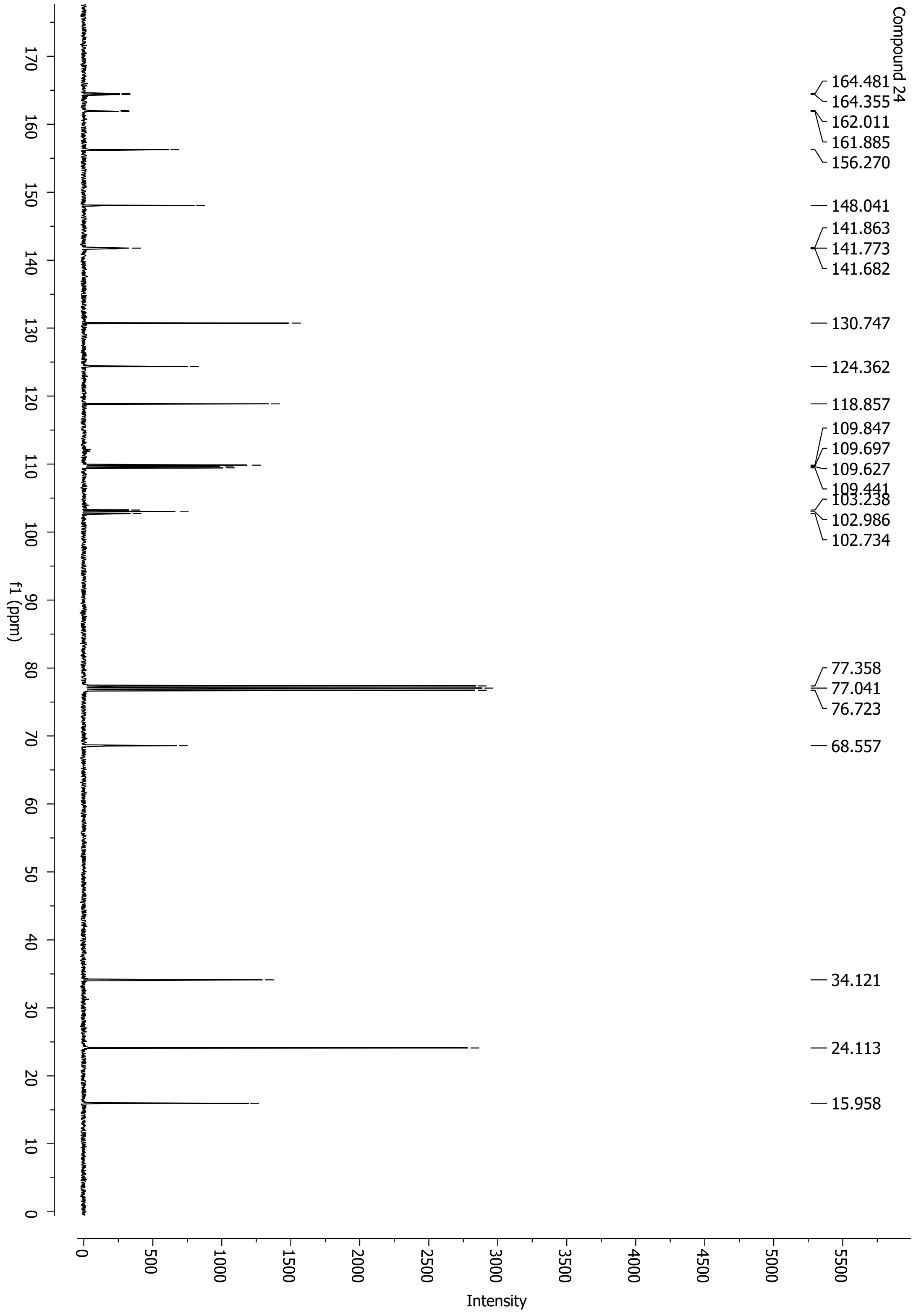

^{1}H spectrum of compound 25

^{13}C spectrum of compound 25

^{19}F spectrum of compound 25

^{1}H-NMR of compound 26

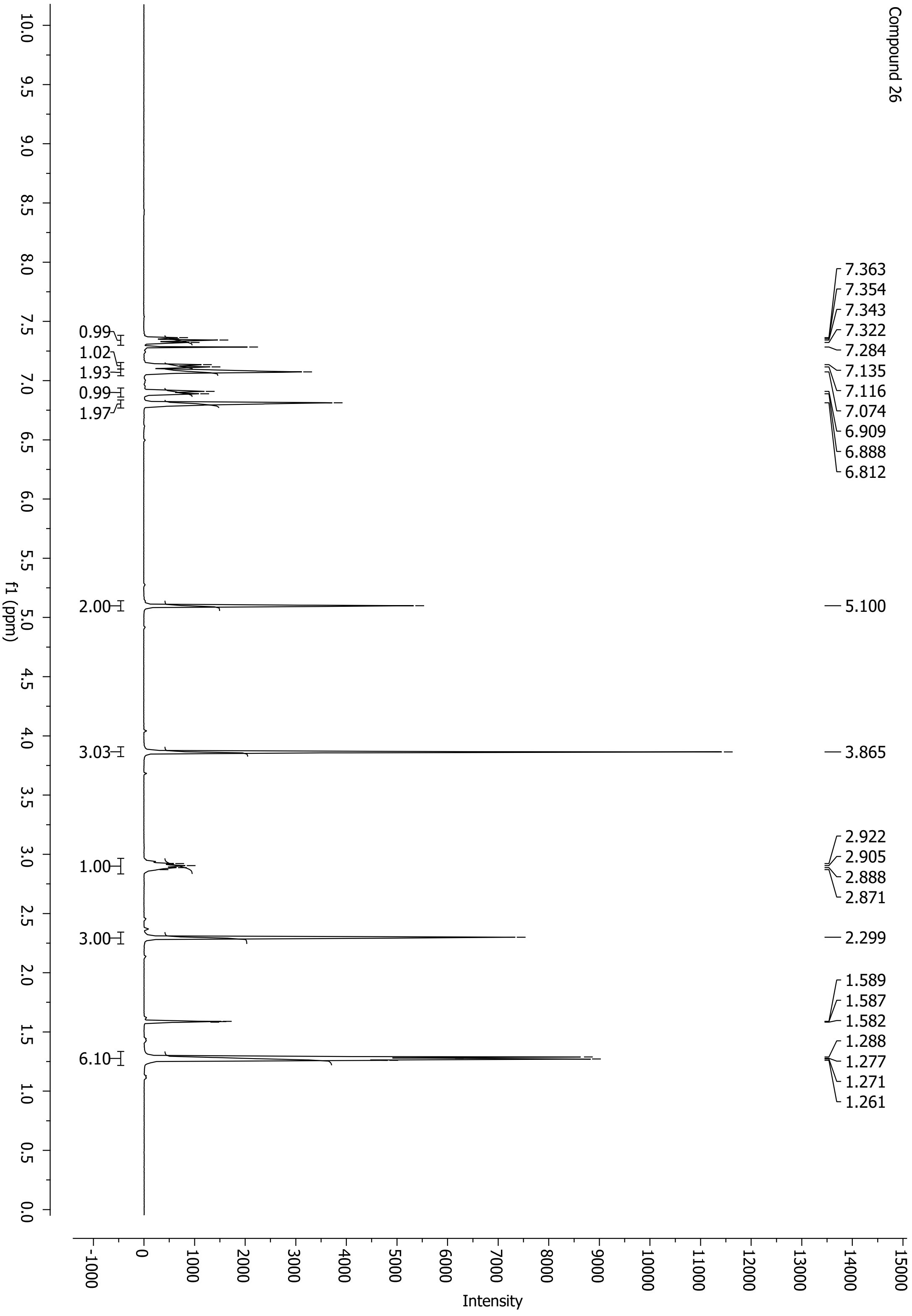

^{13}C-NMR of compound 26

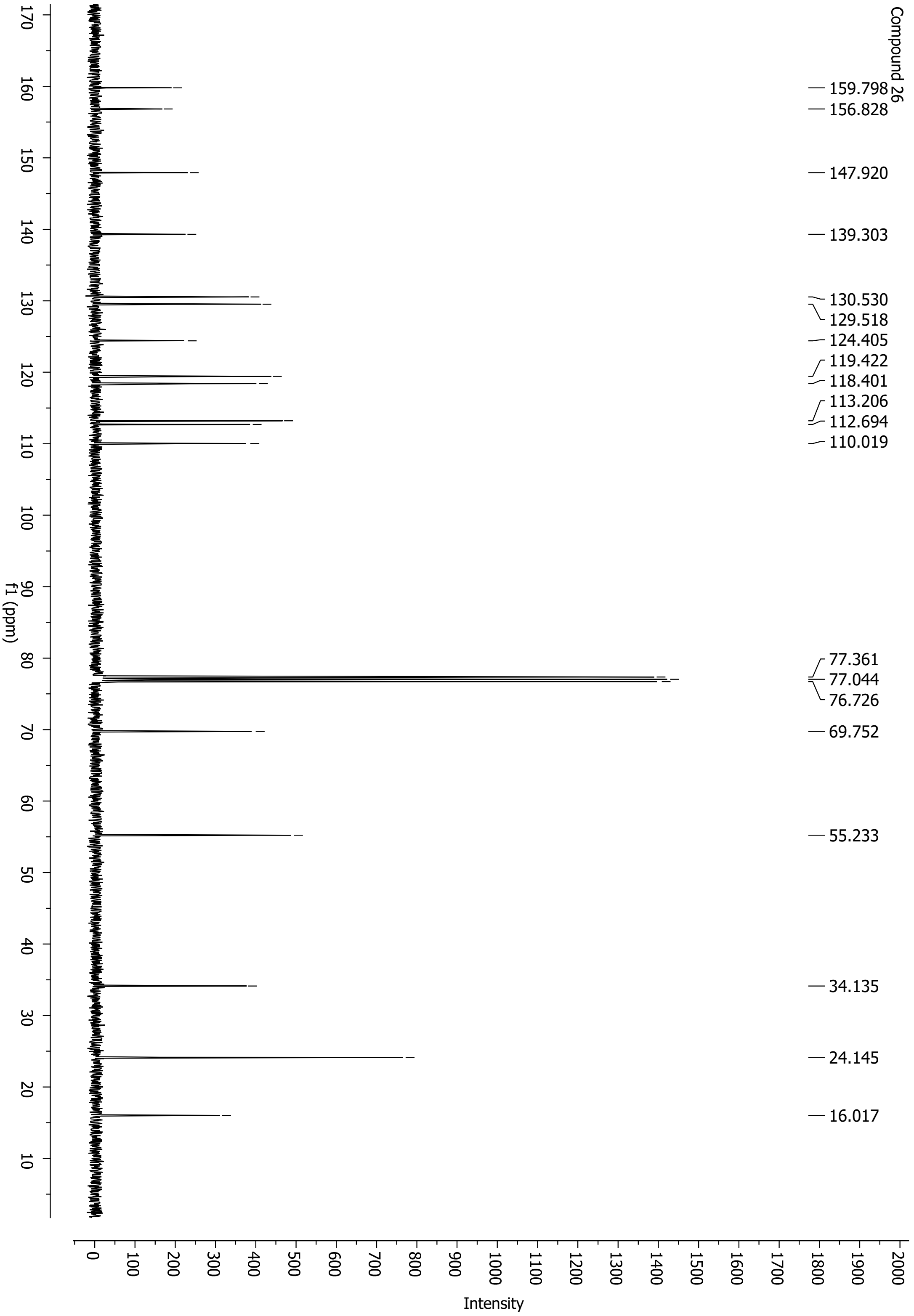

^{1}H spectrum of compound 27

^{13}C spectrum of compound 27

^{1}H-NMR of compound 28

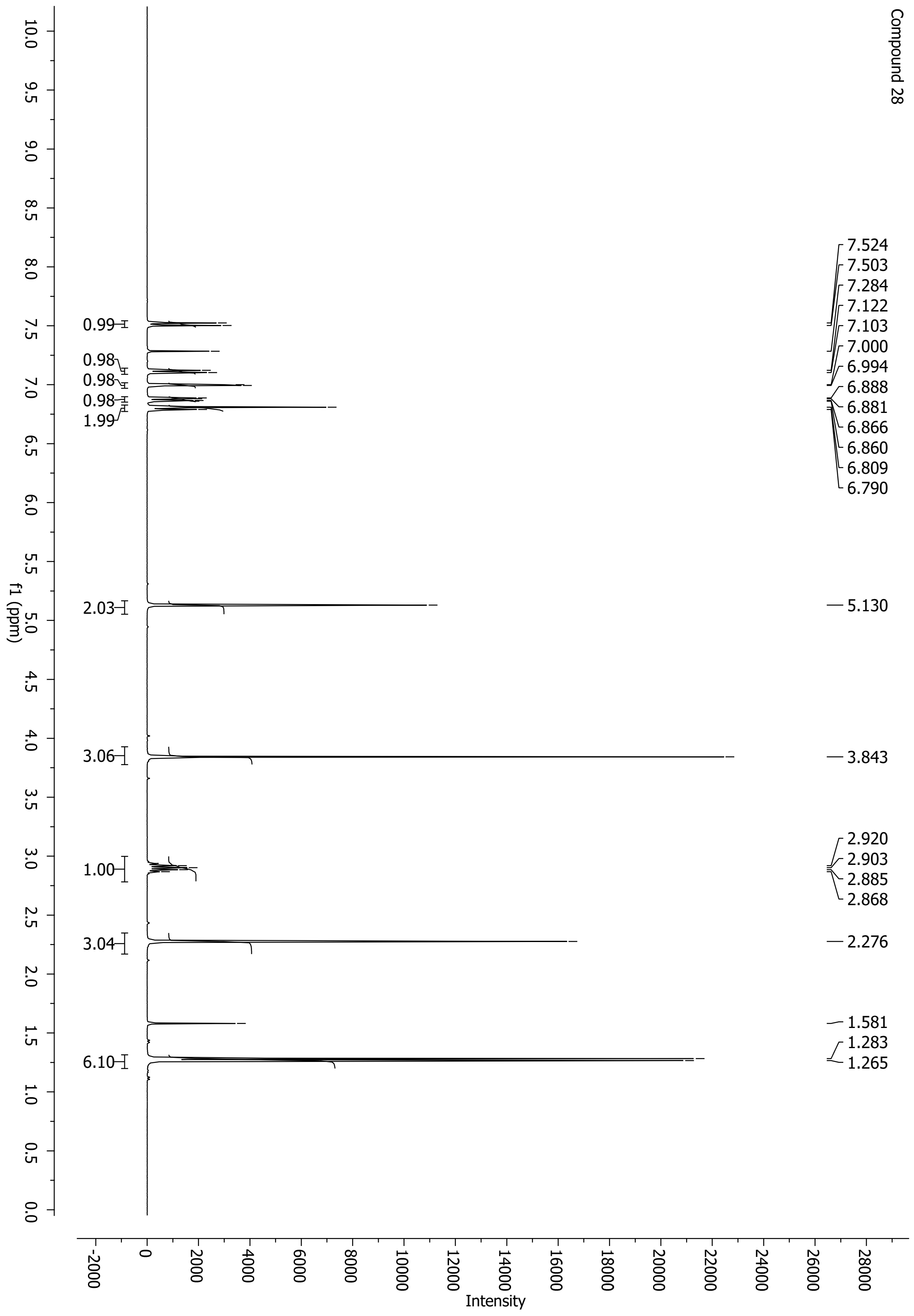

^{13}C-NMR of compound 28

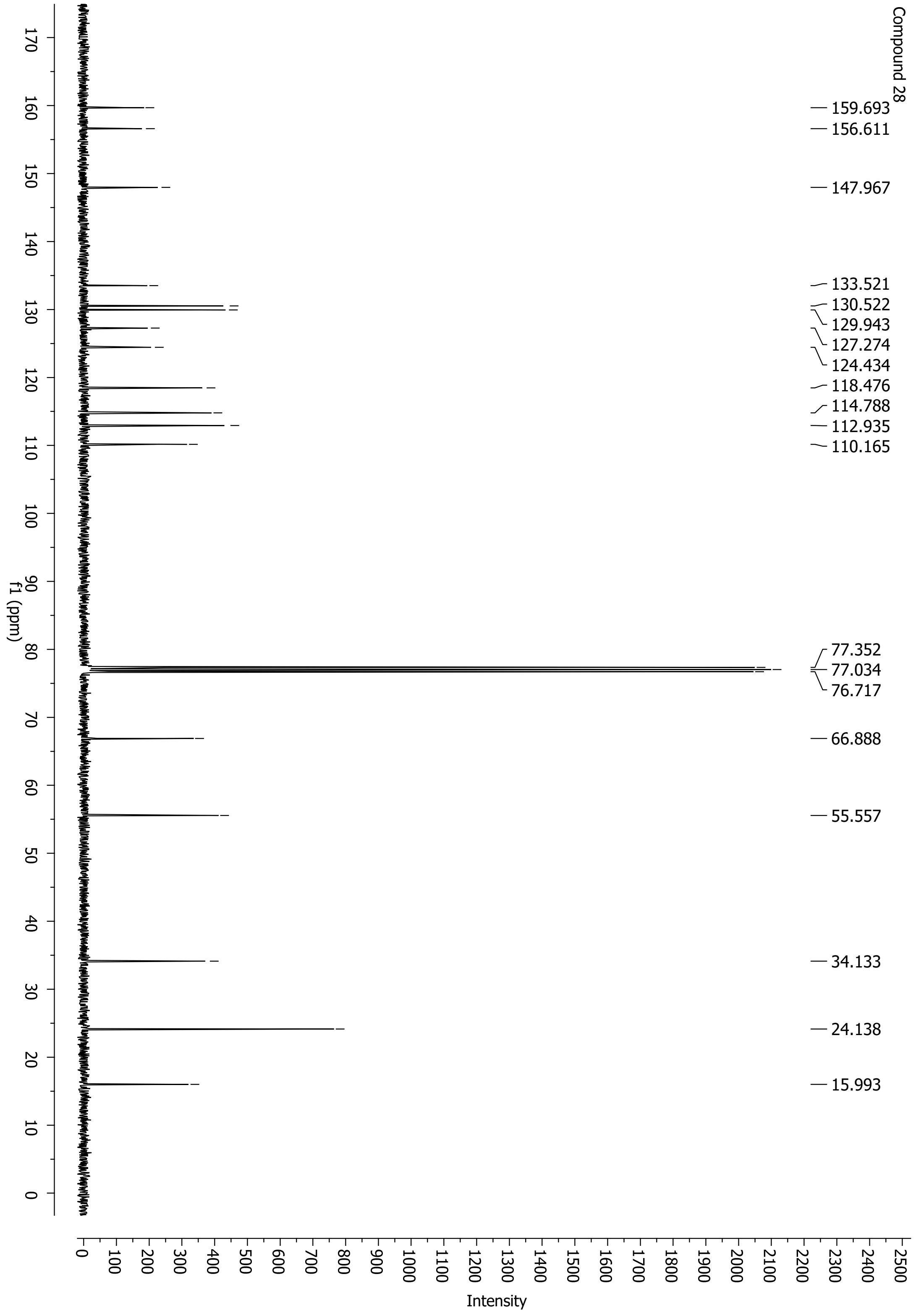

^{1}H spectrum of compound 29

^{13}C spectrum of compound 29

^{1}H spectrum of compound 30

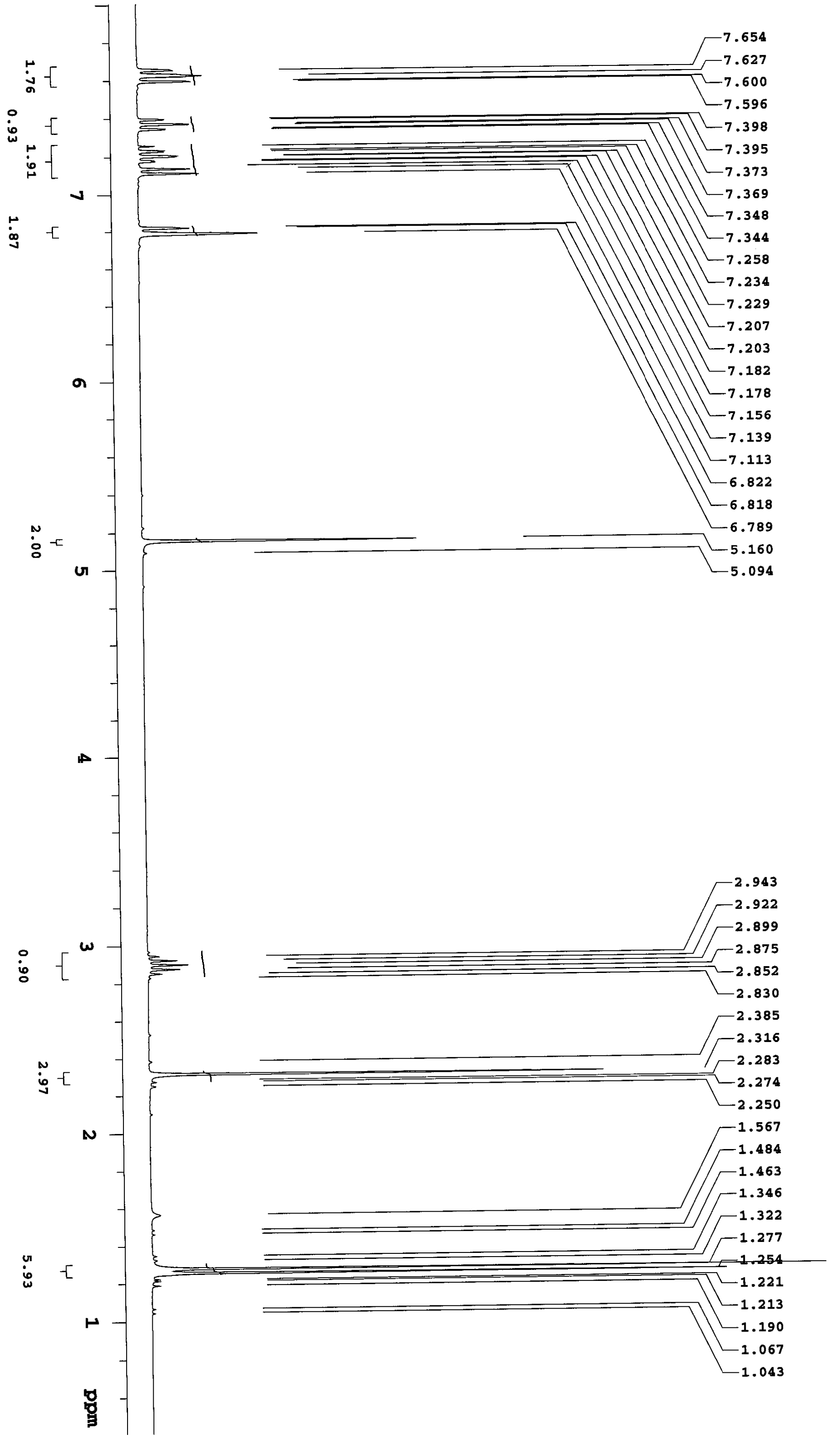

^{13}C spectrum of compound 30

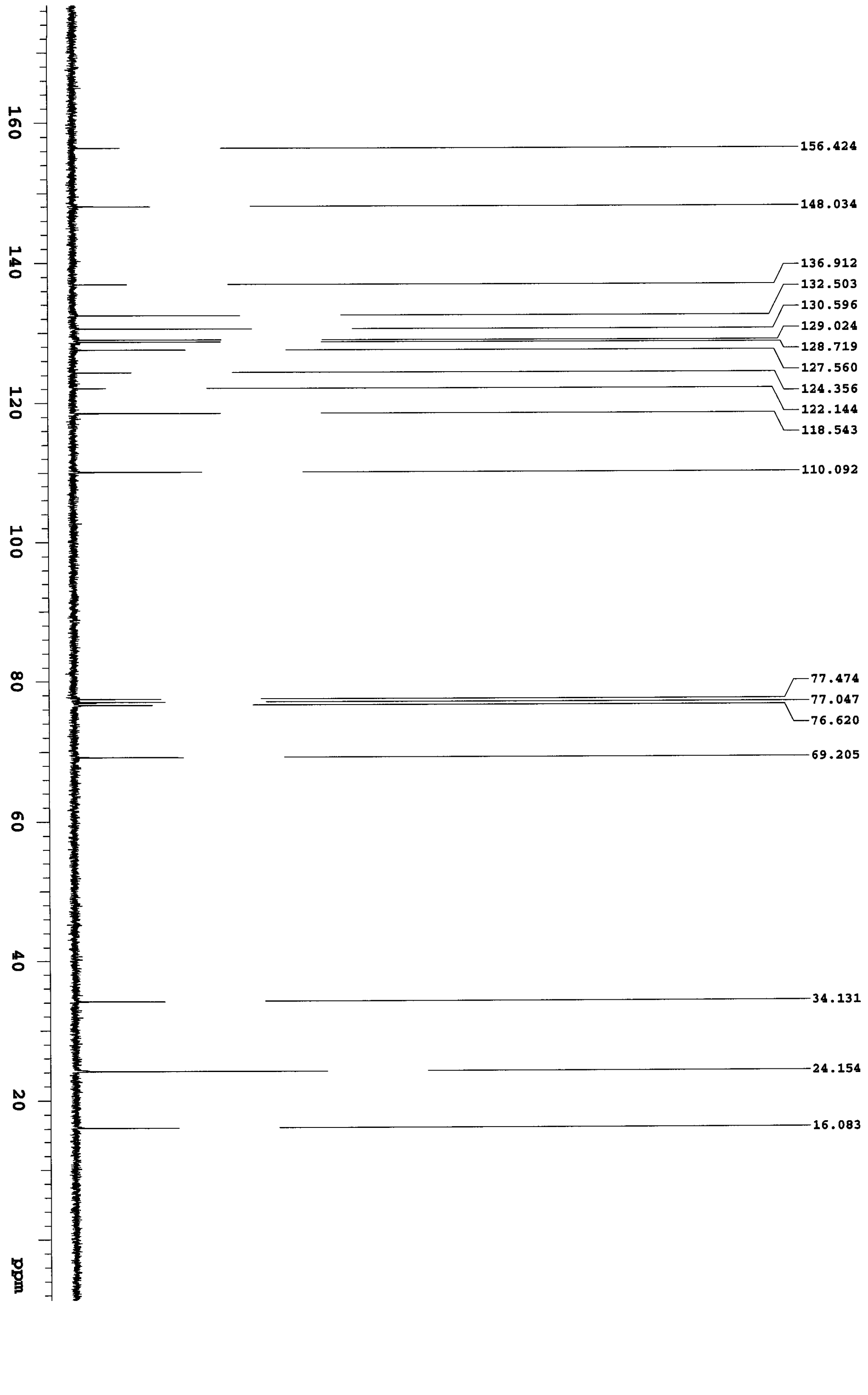

^{1}H spectrum of compound 31

^{13}C spectrum of compound 31

^{1}H spectrum of compound 32

^{13}C spectrum of compound 32

^{1}H spectrum of compound 33

This image cannot currently be displayed.

^{13}C spectrum of compound 33

This image cannot currently be displayed.

^1H spectrum of compound 34

This image cannot currently be displayed.

^{13}C spectrum of compound 34

This image cannot currently be displayed.

^{1}H spectrum of compound 35

This image cannot currently be displayed.

^{1}H spectrum of compound 36

This image cannot currently be displayed.

^{13}C spectrum of compound 36

This image cannot currently be displayed.

^{1}H spectrum of compound 37

This image cannot currently be displayed.

^{13}C spectrum of compound 37

This image cannot currently be displayed.

^{1}H-NMR of compound 40

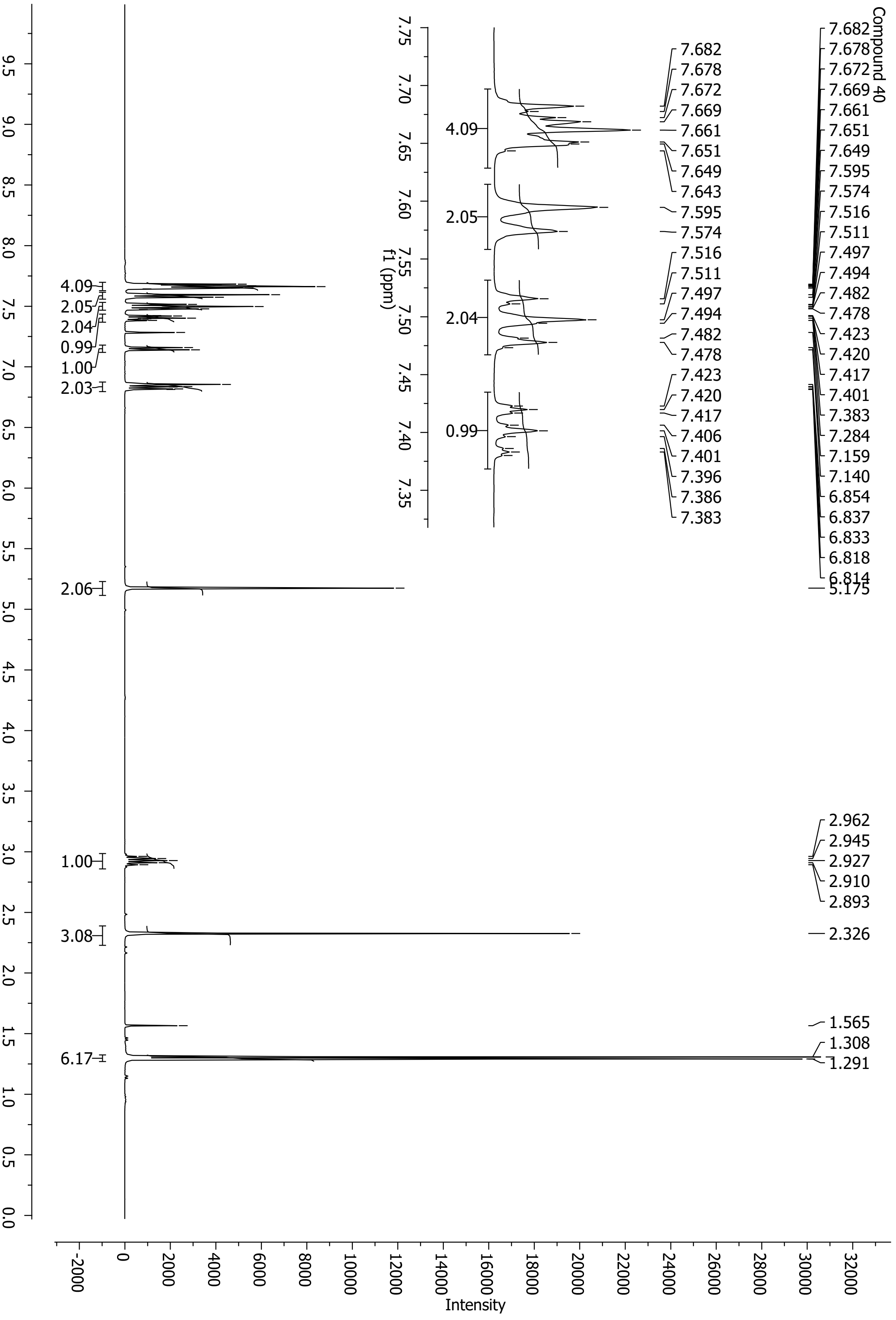

^{13}C-NMR of compound 40

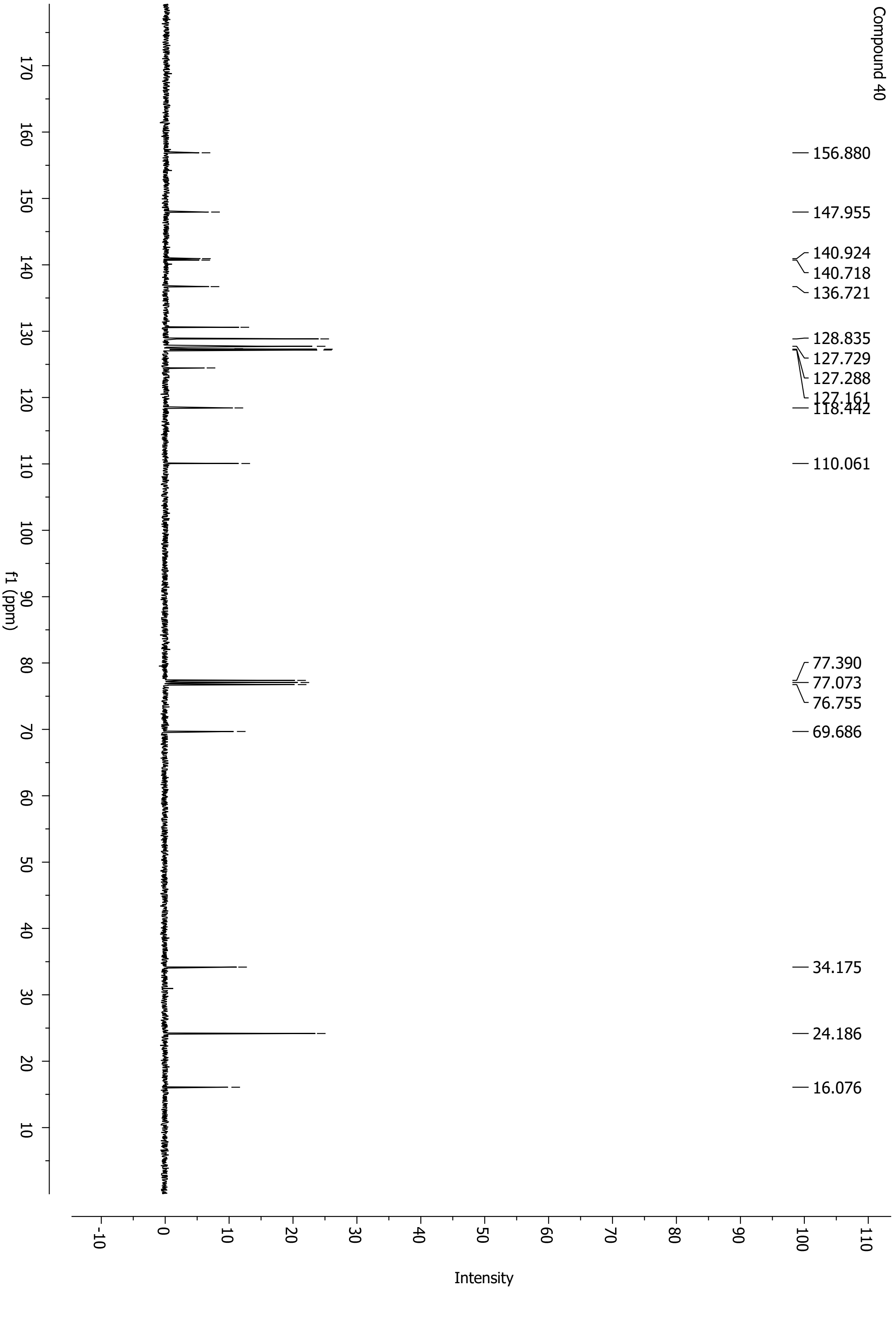

^{1}H spectrum of compound 42

This image cannot currently be displayed.

^{13}C spectrum of compound 42

This image cannot currently be displayed.

^{1}H spectrum of compound 43

This image cannot currently be displayed.

^{13}C spectrum of compound 43

This image cannot currently be displayed.

^{1}H spectrum of compound 44

This image cannot currently be displayed.

^{13}C spectrum of compound 44

This image cannot currently be displayed.

^{1}H spectrum of compound 45

This image cannot currently be displayed.

^{13}C spectrum of compound 45

This image cannot currently be displayed.
